# Rickettsial pathogen augments tick vesicular-associated membrane proteins for infection and survival in the vector host

**DOI:** 10.1128/mbio.03549-24

**Published:** 2025-02-14

**Authors:** Prachi Namjoshi, Jaydeep Kolape, Avni Patel, Hameeda Sultana, Girish Neelakanta

**Affiliations:** 1 Department of Biomedical and Diagnostic Sciences, College of Veterinary Medicine, University of Tennessee, Knoxville, Tennessee, USA; 2 Advanced Microscopy and Imaging Center, College of Arts and Sciences, University of Tennessee, Knoxville, Tennessee, USA; The Ohio State University, Columbus, Ohio, USA

**Keywords:** * Anaplasma phagocytophilum*, rickettsial pathogen, *Ixodes scapularis*, vesicular associated membrane protein (VAMP), pathogen entry, endosome, ticks, tick cells, morulae

## Abstract

*Anaplasma phagocytophilum* is an obligate intracellular rickettsial pathogen that infects humans and animals. The black-legged tick *Ixodes scapularis* acts as a vector and transmits this bacterium to the vertebrate host. Upon entry into a host cell, *A. phagocytophilum* resides and multiplies in a host-derived vacuole called morulae. There is not much information available on the molecules that play an important role(s) in *A. phagocytophilum* entry and formation of these morulae in tick cells. In this study, we provide evidence that tick vesicular-associated membrane proteins, VAMP3 and VAMP4, play important roles in this phenomenon. Quantitative real-time polymerase chain reaction (QRT-PCR) analysis showed that both *vamp3* and *vamp4* transcripts are significantly upregulated at early time points of *A. phagocytophilum* infection in tick cells. We noted that both VAMP3 and VAMP4 predominantly localized to the *A. phagocytophilum*-containing vacuole. RNAi-mediated silencing of *vamp3* and/or *vamp4* expression, followed by confocal microscopy and expression analysis, indicated an impairment in *A. phagocytophilum* morulae formation in tick cells. We also noted that VAMP3 and VAMP4 play a role in the *A. phagocytophilum* persistent infection of ticks and tick cells. Furthermore, RNAi-mediated silencing of expression of arthropod *vamp3* and *vamp4* affected bacterial acquisition from an infected murine host to ticks. Collectively, this study not only provides evidence on the role of arthropod vesicular-associated membrane proteins in *A. phagocytophilum* morulae formation in tick cells but also demonstrates that these proteins are important for bacterial acquisition from an infected vertebrate host into ticks.

**IMPORTANCE:**

*Anaplasma phagocytophilu*m is a tick-borne pathogen primarily transmitted by black-legged Ixodes scapularis ticks to humans and animals. This bacterium enters host cells, forms a host-derived vacuole, and multiplies within this vacuole. The molecules that are critical in the formation of host-derived vacuole in tick cells is currently not well-characterized. In this study, we provide evidence that arthropod vesicular-associated membrane proteins, VAMP3 and VAMP4, are critical for *A. phagocytophilum* early and persistent infection in tick cells. These arthropod proteins are important for the formation of host-derived vacuoles in tick cells. Our study also provides evidence that these proteins are important for *A. phagocytophilum* acquisition from the infected murine host into ticks. Characterization of tick molecules important in bacterial entry and/or survival in the vector host could lead to the development of strategies to target this and perhaps other rickettsial pathogens.

## INTRODUCTION

*Anaplasma phagocytophilum,* a causative agent of human anaplasmosis, primarily infects granulocytes like neutrophils (1–3). The symptoms of human anaplasmosis include malaise, headache, thrombocytopenia, and fever (4). There are no vaccines approved against human anaplasmosis, and early treatment with doxycycline antibiotic is recommended (4–6). Blood smear analysis often visualizes the presence of *A. phagocytophilum* colonies in a host-derived vacuole known as morulae (6, 7). Morulae avoids fusion with the lysosomes and acts like a safe niche for the *A. phagocytophilum* replication, which eventually facilitates establishment of this persistent bacterial infection (8–10).

The black-legged tick, *Ixodes scapularis* (also known as deer tick), is a primary vector for transmission of *A. phagocytophilum* to various vertebrate hosts like humans, deers, cattle, horses, and pets (1, 11, 12). The life cycle of *I. scapularis* consists of four developmental stages—eggs, larvae, nymphs, and adults. *Anaplasma phagocytophilum* infection is transstadially maintained in the tick developmental stages (13).

*Anaplasma phagocytophilum* exists in biphasic developmental stages—dense core (DC) form and reticulate-core (RC) form (14). The DC form is a smaller, compact dense-cored cell, with a dense nucleoid (14). The RC form is larger and has a dispersed nucleoid (7). *Anaplasma phagocytophilum* infects host cells in its DC form. Upon entering the host cell, *A. phagocytophilum* creates a host-derived DC-form-filled vacuole called morulae. Within the morulae, the DC form transforms into the RC form and begins to replicate to establish the infection (1, 8). When the bacterium is ready to escape the infected host cell, the RC form reverts to the DC form (1, 3, 15). *Anaplasma phagocytophilum* is known to establish its infection by delaying apoptosis in host cells (9, 10, 16) by various mechanisms like p38-signaling activation in human neutrophils (17) and in tick cells (18); inhibition of ROS in human and murine neutrophils (19–21) and in tick cells (22), modulation of the tick tryptophan pathway via OATPs (23–26), alteration of several signaling cascades (27–30), and mitigation of metabolic pathways in tick cells (31, 32). Troese *et al.* showed that *A. phagocytophilum* DC form facilitates adhesion to the host cell surface (7). Like *A. phagocytophilum*, other obligate intracellular bacteria like *Chlamydia trachomatis* (33) and *Ehrlichia chaffeensis* (34) also exist in a biphasic developmental cycle.

In the mammalian cells, *A. phagocytophilum*-containing vacuoles are enriched with soluble *N*-ethylmaleimide-sensitive factor attachment protein receptor (SNARE) proteins (35). SNARE proteins are primarily important for the fusion of vesicles with the target membrane, endocytosis, exocytosis, to mediate fusion of vesicles with other membrane-bound cell organelles like lysosomes, and formation of neurotransmitter-containing synaptic vesicles in neurons (36). There are two types of SNAREs—vesicle SNAREs (v-SNAREs), involved in vesicular membranes, and target SNAREs (t-SNAREs), which are associated with nerve terminal membranes (37). Based on arginine or glutamine amino acids positioned with the alpha helix of the SNARE protein, SNAREs could be either classified as R-SNARE or Q-SNARE, respectively (38). R-SNAREs act as v-SNAREs, and Q-SNAREs act as t-SNAREs (39). R-SNAREs are also known as vesicle-associated membrane proteins (VAMPs) (33, 40, 41).

Despite numerous studies that focused on analyzing the dynamics of *A. phagocytophilum* interactions with mammalian cells, very less information is known on how this bacterium enters and forms the intracellular *A. phagocytophilium*-occupied vacuoles in tick cells. In this study, we provide evidence that arthropod SNARE proteins such as VAMP3 and VAMP4 are important for *A. phagocytophilum* entry, formation of morulae, and establishment of infection in tick cells. This study will provide further insights on how obligate intracellular bacteria exploit host SNARE proteins to establish its infection in ticks, establishing SNAREs as an important tool to curb the bacterial foothold.

## RESULTS

### Expression of *vamp3* and *vamp4* levels are upregulated in the early time points of *A. phagocytophilum* infection in tick cells

**Fig 1:**
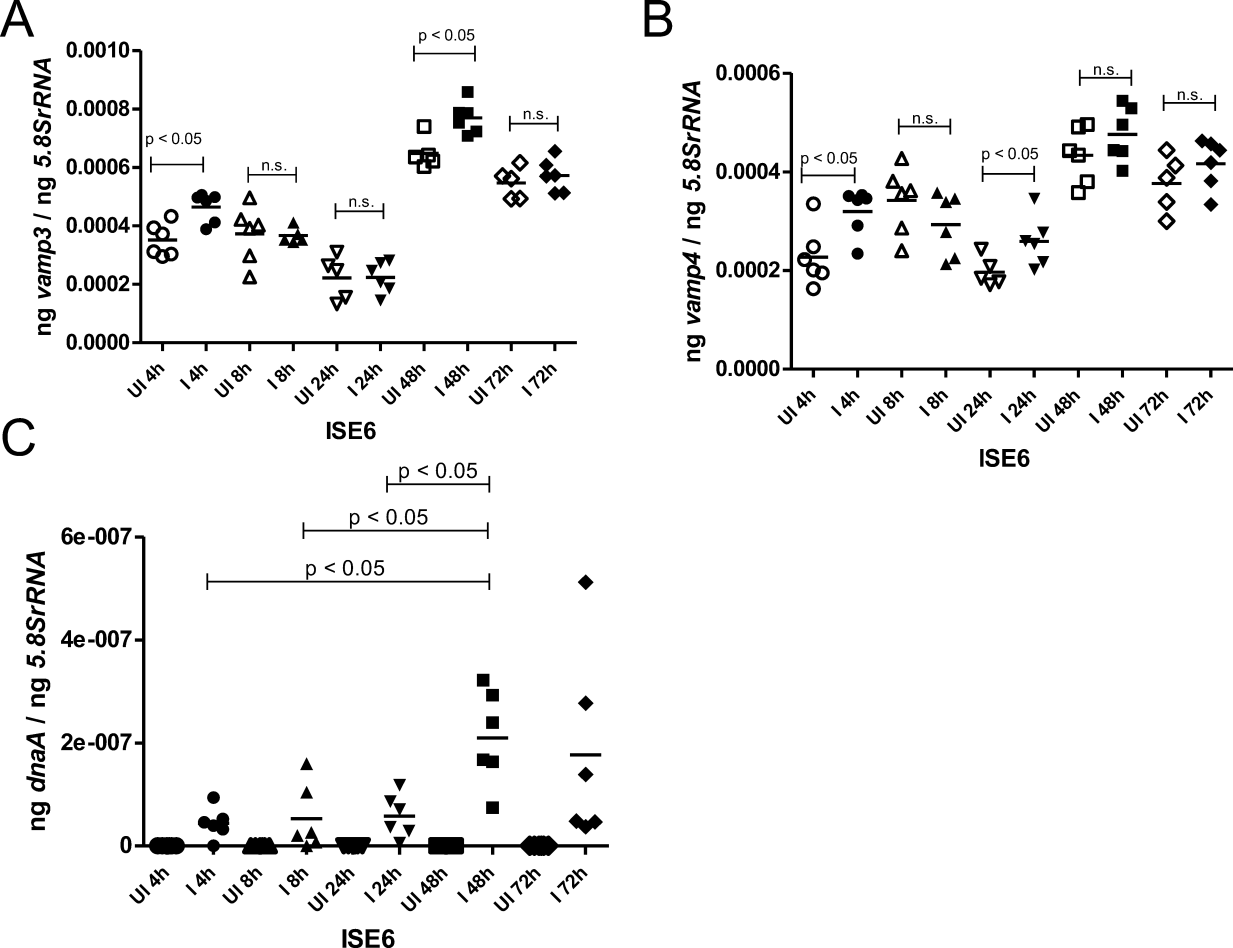
The *vamp3* and *vamp4* transcript levels are upregulated in the early time points of *Anaplasma phagocytophilum* infection in tick cells. QRT-PCR analysis showing expressions of *vamp3* (**A**), *vamp4* (**B**), and bacterial loads (**C**) in uninfected (UI) or *A. phagocytophilum*-infected (**I**) tick cells at different time points post-infection. Each circle represents data from one independent culture plate well. Horizontal lines in the graphs represent the mean of the data points. The mRNA levels of these genes or bacterial *dnaA* levels were normalized to *5.8SrRNA* levels. For panels A and B, *P* value from Student’s *t*-test is shown, and for panel C, *P* value from ANOVA analysis is shown. ng on the *y*-axis indicates nanograms.

A previous study has reported that the human granulocytic ehrlichiosis (HGE) agent enters tick cells by 4 hours post-infection (p.i.) (42). The *Ixodes scapularis* genome encodes several *vamp* genes. We first analyzed to know if there are any changes in the expression of tick VAMPs at an early stage of *A. phagocytophilum* infection in tick cells. We analyzed the transcript levels of several *vamps* including *vamp2*, *vamp3*, *vamp4*, *vamp7,* and *vamp33* at different time points (4 hours, 8 hours, 24 hours, 48 hours, and 72 hours p.i.) upon *A. phagocytophilum* infection in the ISE6 tick cell line. QRT-PCR analysis revealed that the expression of *vamp3* was significantly (*P* < 0.05) increased at 4 hours and 48 hours post-infection in *A. phagocytophilum*-infected tick cells when compared to the levels noted in uninfected controls (Fig. 1A). We also observed significantly (*P* < 0.05) increased gene expression of *vamp4* at 4 hours and 24 hours post-infection in *A. phagocytophilum*-infected tick cells when compared to the levels noted in uninfected controls (Fig. 1B). The transcripts of *vamp2* were unaltered upon *A. phagocytophilum* infection at all tested time points (Fig. S1A). At 24 hours p.i., *vamp7* and *vamp33* transcripts were significantly (*P* < 0.05) downregulated in *A. phagocytophilum*-infected tick cells compared to the levels noted in uninfected controls (Fig S1B and C). However, no significant differences were observed in *vamp7* or *vamp33* transcript levels between *A. phagocytophilum*-infected tick cells and uninfected controls at other tested time points (Fig S1B and C). We noted that *A. phagocytophilum* burden was significantly (*P* < 0.05) higher at 48 hours p.i. compared to the bacterial burden observed at 4 hour, 8 hour, and 24 hour p.i. time points (Fig. 1C). The significantly (*P* < 0.05) increased expression of *vamp3* and *vamp4* at 4 hours post-infection indicates a role for these proteins in the early phase of *A. phagocytophilum* infection in tick cells. Therefore, further studies were pursued on these two tick molecules.

### Expressions of *vamp3* and *vamp4* are developmentally regulated

**Fig 2:**
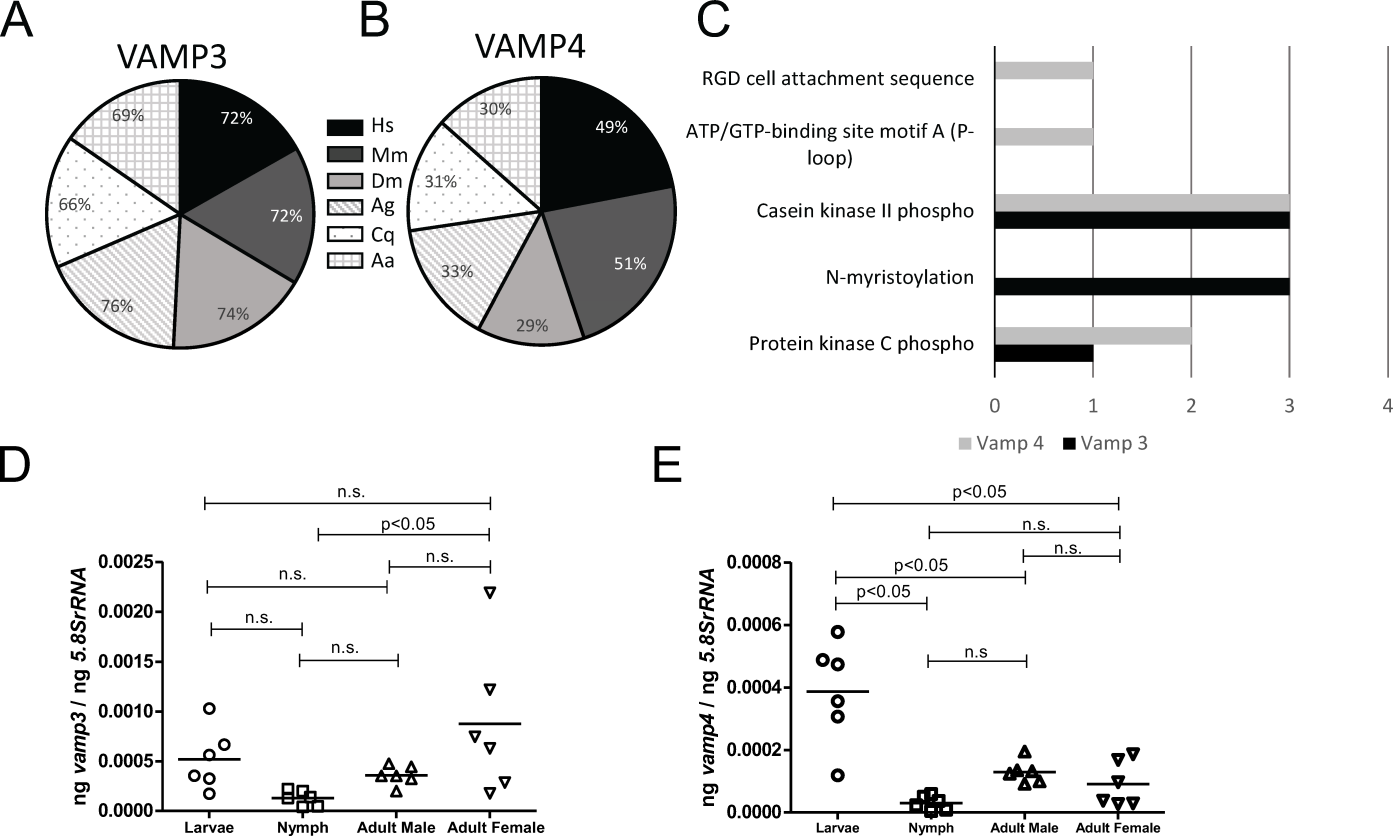
VAMP3 *and* VAMP4 *s*equence analysis and expression at different developmental stages of *I. scapularis*. Pie charts represent the percent identity of *I. scapularis* VAMP3 (**A**) and VAMP4 (**B**) amino acid sequences with ortholog proteins from *Homo sapiens* (Hs)*, Mus musculus* (Mm)*, Drosophila melanogaster* (Dm)*, Anopheles gambiae* (Ag)*, Culex quinquifasciatus* (Cq), and *Aedes aegypti* (Aa). (**C**) Number of predicted post-translational sites in VAMP3 and VAMP4 proteins were analyzed at PROSITE. QRT-PCR analysis showing expression of *vamp3* (**D**) and *vamp4* (**E**) transcripts at different tick developmental stages (larvae, nymphs, males, and females) in uninfected unfed ticks. Each data point in nymphs and adult samples represents transcript levels noted in an individual tick. For larval samples, each data point represents transcript levels noted in five pooled larvae. Horizontal lines in the graphs represent the mean of the data points. The mRNA levels are normalized to *5.8SrRNA* levels. Statistical significance was calculated using ANOVA. ng on the *Y*-axis indicates nanograms.

Percent identity values of the *I. scapularis* VAMP3 (Fig. 2A) and VAMP4 (Fig. 2B) amino acid sequences with ortholog proteins from *Homo sapiens* (Hs)*, Mus musculus* (Mm)*, Drosophila melanogaster* (Dm)*, Anopheles gambiae* (Ag)*, Culex quinquifasciatus* (Cq), and *Aedes aegypti* (Aa) were determined by CLUSTALW alignment. The *Ixodes scapularis* VAMP3 amino acid sequence shares 72%, 72%, 74%, 76%, 66%, and 69% percent identity with *Homo sapiens* (human)*, Mus musculus* (mouse), *D. melanogaster, A. gambiae, C. quinquifasciatus,* and *A. aegypti* VAMP3 orthologs, respectively (Fig. 2A; Fig. S2A and S3A). The *Ixodes scapularis* VAMP4 amino acid sequence shares 49%, 51%, 29%, 33%, 31%, and 30% percent identity with human, mouse, *D. melanogaster, A. gambiae, C. quinquifasciatus,* and *A. aegypti* VAMP4 orthologs, respectively (Fig. 2B; Fig. S2B and S3B). Bioinformatic analysis of post-translational modification sites in *I. scapularis* VAMP3 and VAMP4 revealed that both these proteins have one or two protein kinase C phospho and three casein kinase II phospho sites, respectively (Fig. 2C). In addition, *I. scapularis* VAMP4 has one each of RGD cell attachment sequence and ATP/GTP-binding site motif, and VAMP3 has three N-myristoylation sites (Fig. 2C). Phylogenetic tree analysis showed that *I. scapularis* VAMP3 forms a clade close to human and mice orthologs (Fig. S4A), whereas VAMP3 orthologs from mosquitoes form a separate clade (Fig. S4A). In addition, the phylogenetic analysis revealed that tick VAMP4 forms a clade close to human and mice proteins and *D. melanogaster* and mosquito proteins form a different clade (Fig. S4B). Furthermore, *I. scapularis* VAMP3 shares around 78%–89% and VAMP4 shares between 25% and 97% identity with orthologs from *Amblyomma americanum, Dermacentor andersoni, Ixodes hexagonus,* and *Rhipicephalus sanguineus* tick sequences (Fig. S5 and 6). Phylogenetic analysis revealed that *I. scapularis* VAMP3 and VAMP4 proteins fall in the same clade with *I. hexagonus* orthologs (Fig. S6C and D). We then analyzed the mRNA expression levels of *vamp3 and vamp4* in tick developmental stages (Fig. 2D and E). Quantitative real-time polymerase chain reaction analysis showed that nymphs expressed significantly (*P* < 0.05) lower levels of *vamp3* (Fig. 2D) in comparison to female ticks. No significant (*P* > 0.05) differences in the *vamp3* mRNA levels were observed between larvae, male, and female ticks (Fig. 2D). Also, we noted significantly (*P* < 0.05) increased expression of *vamp4* transcripts in larvae compared to the levels noted in nymphs and adult ticks (Fig. 2E). However, no significant (*P* > 0.05) differences in the *vamp4* mRNA levels were observed between nymph, male, and female ticks (Fig. 2E). These data show significantly (*P* < 0.05) variable expression patterns of *vamp3* and *vamp4* transcript levels at different developmental stages of ticks.

### VAMP3 and VAMP4 localize on *A. phagocytophilum*-containing vacuoles

**Fig 3:**
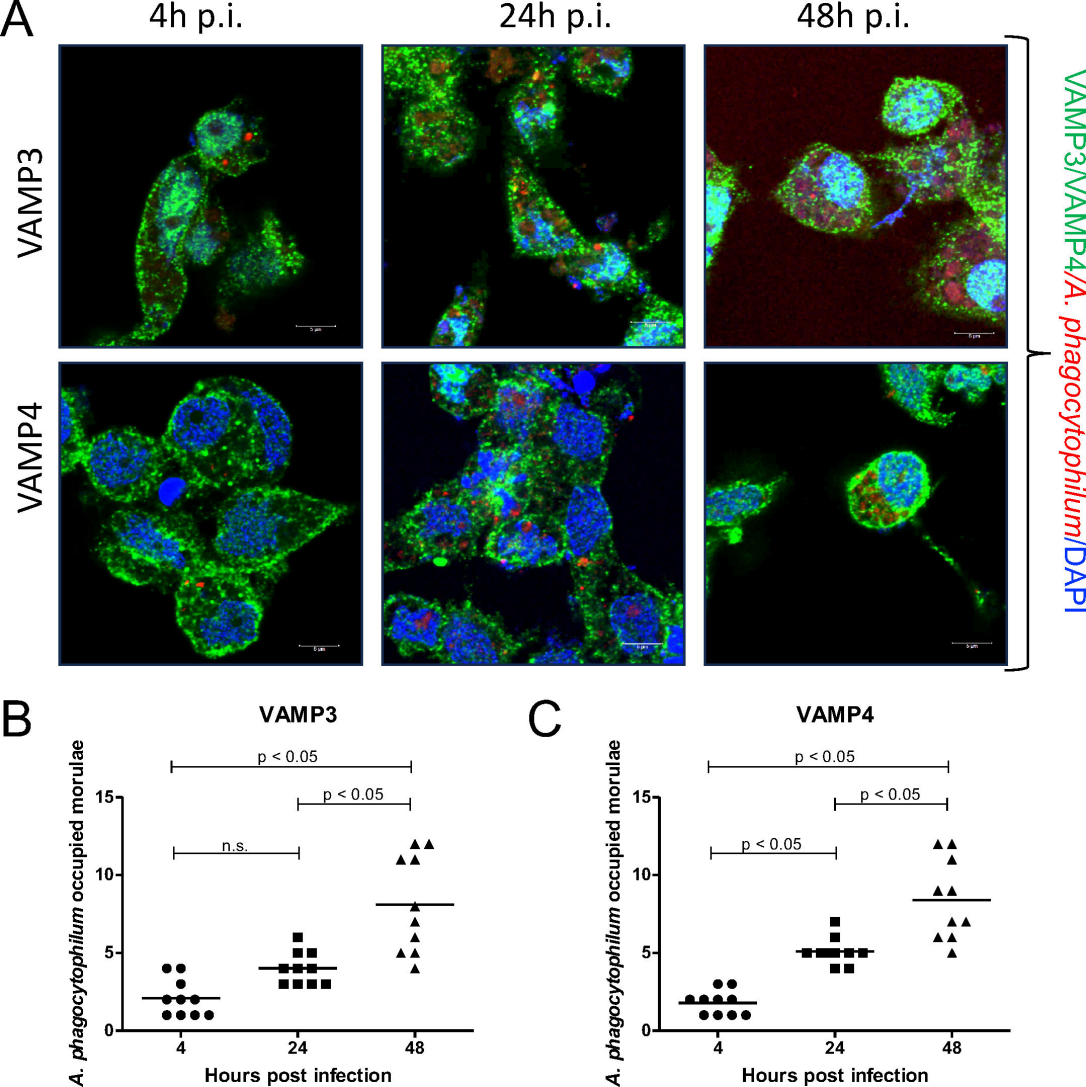
Quantitative and microscopic assessment of VAMP3- or VAMP4-positive *A. phagocytophilum*-containing vacuoles in tick cells**. A**) Confocal microscopic images of mcherry-*A. phagocytophilum* (red)-infected tick cells (4 hours, 24 hours, and 48 hours post-infection) probed with anti-VAMP3 of anti-VAMP4 antibody, followed by the incubation with Alexa Fluor 488-conjugated secondary antibody (green). Cells were also stained with DAPI to visualize nuclei (blue). Original magnification, ×63. Scale bar: 5 µm. **B**) Quantitative analysis showing a number of *A. phagocytophilum*-occupied VAMP3-positive morulae (**B**) and *A. phagocytophilum*-occupied VAMP4-positive morulae (**C**) in tick cells at 4 hours, 24 hours, and 48 hours post-infection. Each circle represents VAMP3- or VAMP4-positive morulae filled with *A. phagocytophilum* in one tick cell. Horizontal lines in the graphs represent mean of the data points. Statistical significance was calculated using ANOVA.

Since we observed the upregulation of *vamp3* at 4 hours and 48 hours p.i. and of *vamp4* at 4 hours and 24 hours p.i, we first examined if VAMP3 and VAMP4 proteins are associated with the *A. phagocytophilum*-containing vacuoles. We first performed an immunofluorescence assay to examine the localization of VAMP3 and VAMP4 to the *A. phagocytophilum*-containing vacuoles in tick cells at different post-infection time points (4 hours, 24 hours, and 48 hours). We noted accumulation of VAMP3 and VAMP4 around *A. phagocytophilum*-containing vacuoles at all tested time points (Fig. 3A). We noted increased number of morulae over time of post-infection from 4 hours to 48 hours (Fig. 3A). Furthermore, quantification of morulae revealed a statistically significant (*P* < 0.05) increase in VAMP3- or VAMP4-positive *A. phagocytophilum*-containing morulae over the time of post-infection from 4 hours to 48 hours (Fig. 3B and C).

**Fig 4:**
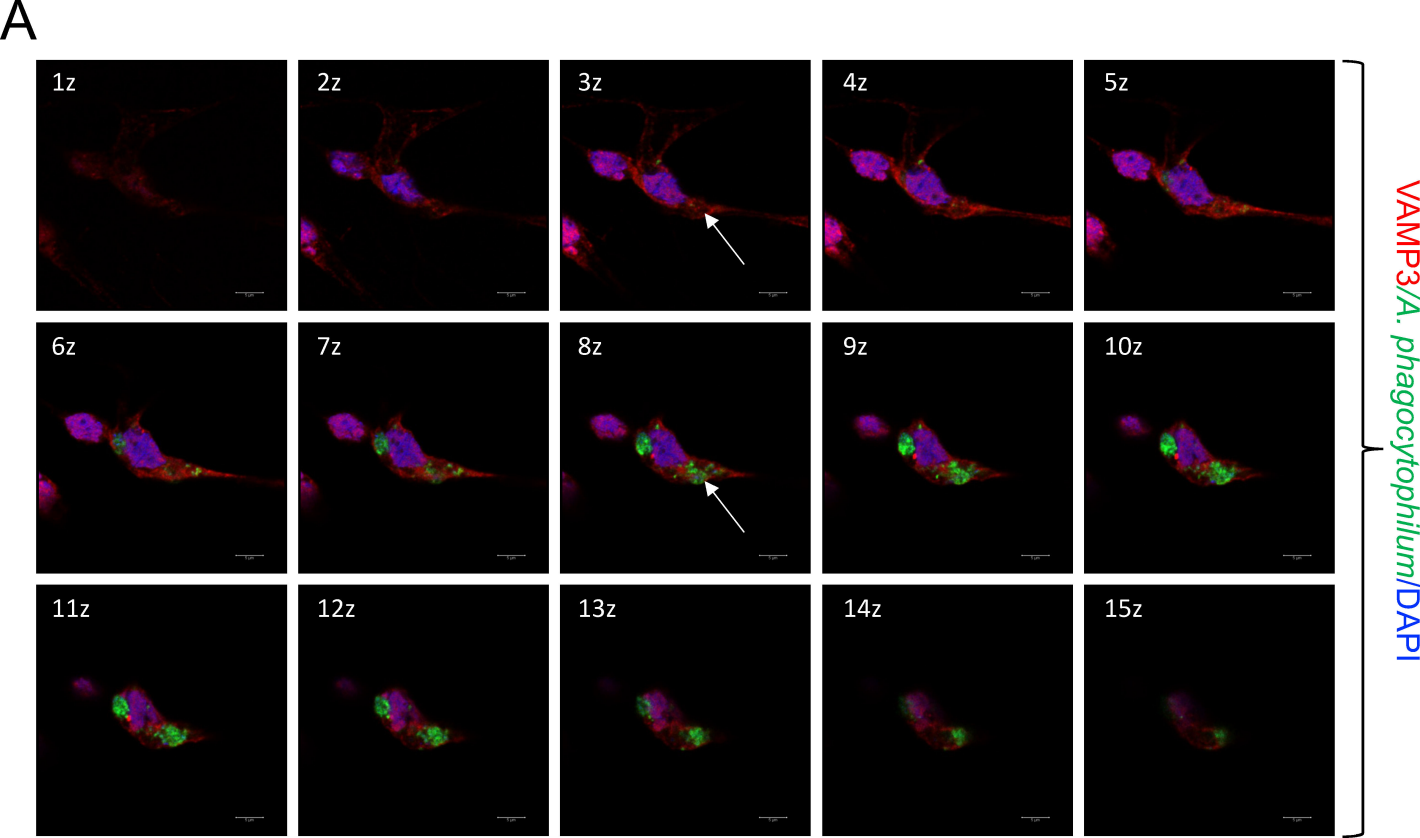
Time-lapse microscopic analysis showing VAMP3 colocalization with *A. phagocytophilum-*containing vacuoles in tick cells. Z-stack cell section images taken 1 z apart (every 0.5 µm of depth) from the Z-stack video (Video S1) are shown. In the 3 z image, a white arrow is pointed at an empty vacuole, whose periphery is decorated with VAMP3. The same empty vacuole appears to be filled with GFP-*A. phagocytophilum* in the 8 z cell section image. Scale bar: 5 µm.

**Fig 5:**
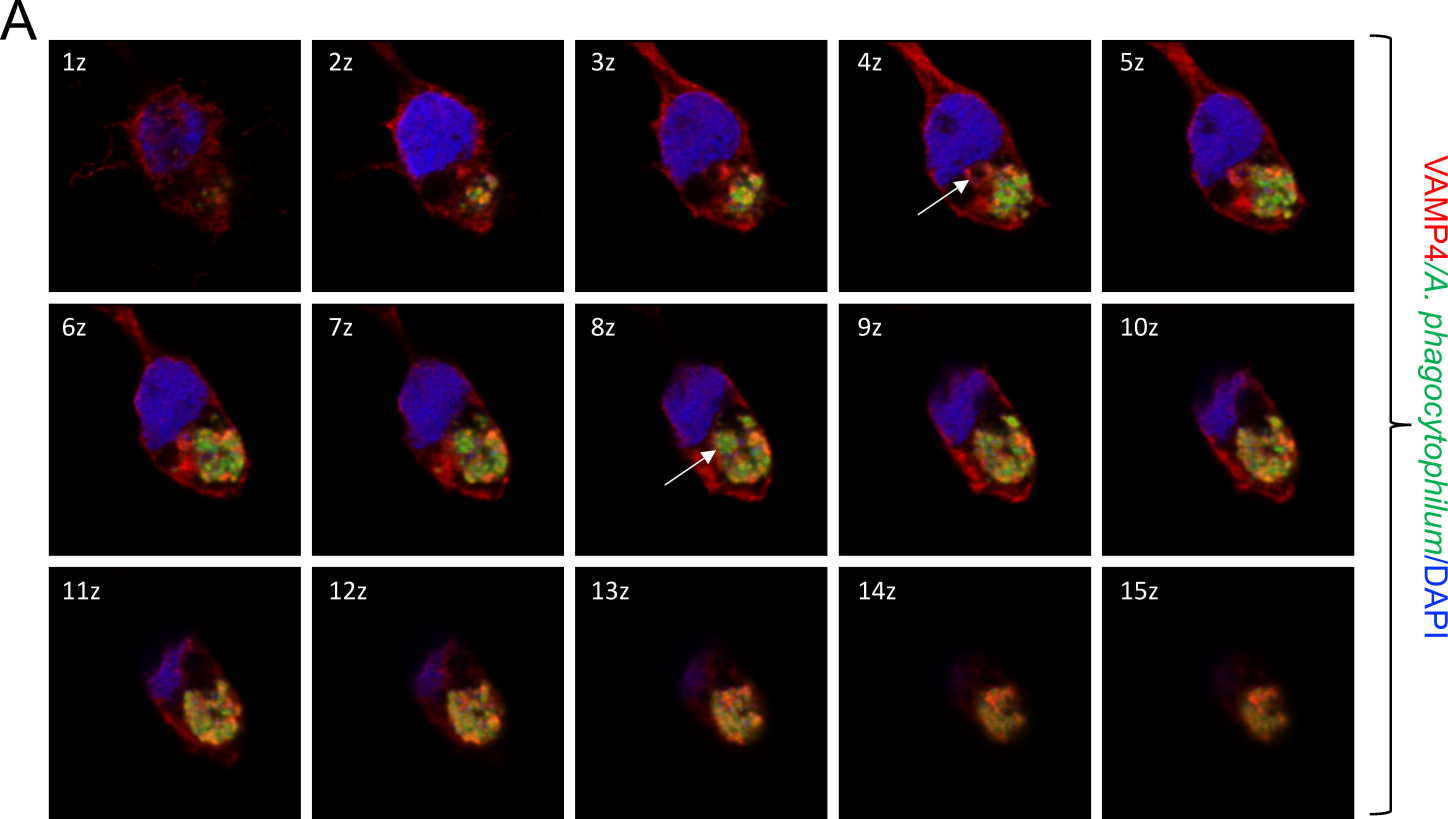
Time-lapse microscopic analysis showing VAMP4 colocalization with *A. phagocytophilum-*containing vacuoles in tick cells. Z-stack cell section images taken 1 z apart (every 0.5 µm of depth) from the Z-stack video (Video S3) are shown. In the 4 z image, a white arrow is pointed at an empty vacuole, whose periphery is decorated with VAMP4. The same empty vacuole appears to be filled with GFP-*A. phagocytophilum* in the 8 z cell section image. Scale bar: 5 µm.

We then performed VAMP3 and VAMP4 localization studies in tick cells that were persistently infected with GFP-tagged *A. phagocytophilum*. We generated a Z-stack video (Video S1) and 3D imaging video (Video S2) of fixed cells showing the colocalization of *A. phagocytophilum*-containing vacuoles with VAMP3 distribution in different focal planes. In Fig. 4, we demonstrate VAMP3 localization in the Z-stack cell section images taken 1 z apart (every 0.5 µm of depth) from Z-stack video (Video S1). As indicated in the 3 z image section, an empty vacuole was observed to be decorated with VAMP3. The same empty vacuole later appears to be filled with GFP-tagged *A. phagocytophilum* in the 8 z section image. Similarly, a Z-stack video (Video S3) and 3D imaging video (Video S4 ) depict the colocalization of *A. phagocytophilum*-containing vacuoles with VAMP4 (Fig. 5). In Fig. 5, we demonstrate VAMP4 localization in the Z-stack cell section images taken 1 z apart (every 0.5 µm of depth) from the Z-stack video (Video S3). As indicated in the 4 z cell section image, an empty vacuole was observed to be decorated with VAMP4. At the 8 z section image, the same empty vacuole now appears to be filled with GFP-tagged *A. phagocytophilum*. Collectively, these microscopic observations show that VAMP3 and VAMP4 localize on *A. phagocytophilum*-containing vacuoles.

### RNAi-mediated silencing of *vamp3* and *vamp4* expression affects *A. phagocytophilum* morulae formation in tick cells

**Fig 6:**
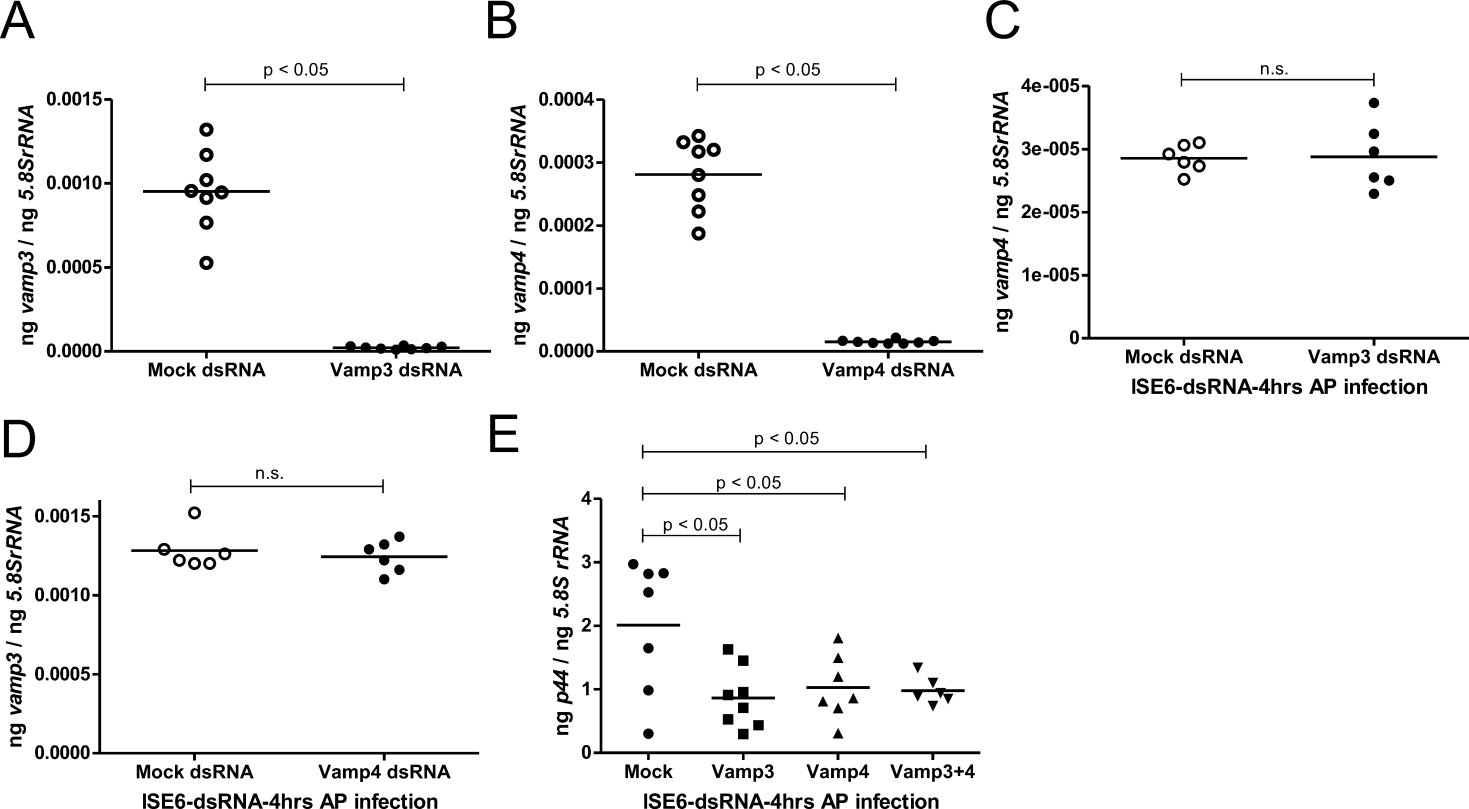
RNAi-mediated silencing of *vamp3* and/or *vamp4* affects the early phase of *A. phagocytophilum* infection in tick cells. QRT-PCR analysis showing expression of *vamp3* (**A**) and *vamp4* (**B**) in *A. phagocytophilum-*infected mock-, or *vamp3*- or *vamp4-*dsRNA-treated tick cells at 4 hours p.i., respectively. QRT-PCR analysis showing *vamp4* transcripts in mock or *vamp3*-dsRNA-treated (**C**) or *vamp3* transcripts in mock or *vamp4*-dsRNA-treated (**D**) *A. phagocytophilum-*infected cells (**E**) *p44* (*A. phagocytophilum* burden) levels in mock- or *vamp3*-, *vamp4*-, or *vamp3 + vamp4-*dsRNA-treated tick cells are shown. Each data point represents data from one independent culture plate well. Horizontal lines in the graphs represent the mean of the data points. The mRNA levels of these genes were normalized to *5.8S rRNA* levels. *P* value from Student’s *t*-test is shown. ng on the *Y*-axis indicates nanograms.

The localization of VAMP3 and VAMP4 on the *A. phagocytophilum*-containing vacuole in cells with persistent bacterial infection prompted us to further investigate whether these proteins are important for the early phase of bacterial infection. We silenced *vamp3* or *vamp4* expression either individually or as combined via RNAi and analyzed *A. phagocytophilum* levels in tick cells. Transfection with mock-, *vamp3*-, or *vamp4*-dsRNAs either individually or in combination did not affect the morphology of tick cells at 24 hours post-dsRNA-transfection and at the 4-hour p.i. time point (Fig. S7). QRT-PCR analysis revealed significant reduction of *vamp3* mRNA (Fig. 6A) and *vamp4* mRNA (Fig. 6B) in the *A. phagocytophilum*-infected *vamp3*-dsRNA- or *vamp4*-dsRNA-treated tick cells in comparison to the mock-treated controls, respectively (Fig. 6A and B). We did not see any differences in the *vamp4* transcripts (Fig. 6C) or *vamp3* transcripts (Fig. 6D) between mock-treated or *vamp3*-dsRNA-treated (Fig. 6C) or *vamp4*-dsRNA-treated (Fig. 6D) tick cells, respectively. However, we also noted a significant reduction in *A. phagocytophilum* burden in *vamp3*-dsRNA- or *vamp4*-dsRNA treated tick cells when compared to the bacterial burden observed in mock-treated controls (Fig. 6E). QRT-PCR analysis upon combined silencing of both *vamp3* and *vamp4* transcripts also revealed a significant reduction in bacterial burden in *vamp3 + vamp4*-dsRNA-treated tick cells when compared to the bacterial burden noted in mock-treated controls (Fig. 6E).

To further support the QRT-PCR data, we performed confocal microscopy for detailed analysis. Tick cells were infected with GFP-tagged *A. phagocytophilum,* and immunofluorescence analysis was performed at 24 hours post-dsRNA-transfection and at the 4-hour p.i. time point. The microscopic analysis revealed that expression of VAMP3 and VAMP4 was profoundly diminished in *vamp3*- or *vamp4*-dsRNA-treated GFP-tagged *A. phagocytophilum*-infected tick cells, respectively, when compared to their respective mock-treated controls (Fig. S8A and B). In addition, we observed a decreased in the absence of bacterial signals in *vamp3*- or *vamp4*-silenced tick cells when compared to the levels noted in respective mock-treated controls (Fig. S8A and B). Taken together, these results show that the expressions of VAMP3 and VAMP4 are important during the initial phase of *A. phagocytophilum* infection in tick cells.

### RNAi-mediated silencing of *vamp3* and *vamp4* expressions affects *A. phagocytophilum* survival in tick cells

**Fig 7:**
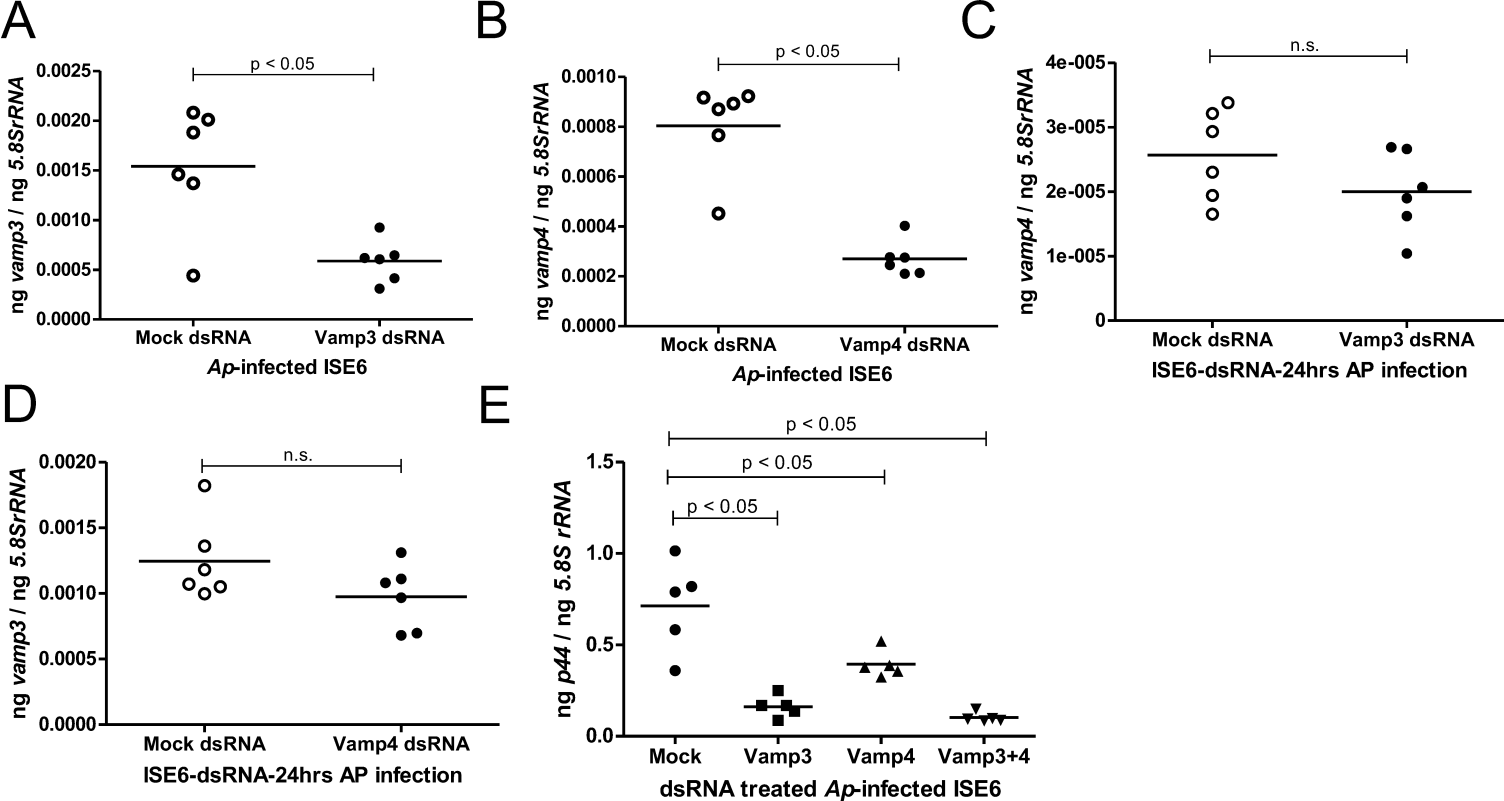
RNAi-mediated silencing of *vamp3* and *vamp4* affects later phases of *A. phagocytophilum* infection in tick cells. QRT-PCR analysis showing expressions of *vamp3* (**A**) and *vamp4* (**B**) in *A. phagocytophilum-*infected mock-, *vamp3*-, or *vamp4-*dsRNA-treated tick cells at 24 hours p.i., respectively. QRT-PCR analysis showing *vamp4* transcripts in mock or *vamp3*-dsRNA-treated (**C**) or *vamp3* transcripts in mock or *vamp4*-dsRNA-treated (**D**) *A. phagocytophilum-*infected cells. (**E**) *p44* (*A. phagocytophilum* burden) levels in mock-, *vamp3*-, *vamp4*-, or *vamp3 + vamp4-*dsRNA-treated tick cells are shown. Each data point represents data from one independent culture plate well. Horizontal lines in the graphs represent mean of the data points. The mRNA levels of these genes were normalized to *5.8S rRNA* levels. *P* value from Student’s *t*-test is shown. ng on the *Y-*axis indicates nanograms.

We further explored if silencing of *vamp3* and/or *vamp4* affects *A. phagocytophilum* survival at later time points post-infection. Transfection with mock-, *vamp3*-, and/or *vamp4*-dsRNAs did not affect the morphology of *A. phagocytophilum*-infected tick cells at 24 hours post-transfection and at the 24-hour p.i. time point compared to the mock-treated controls (Fig. S9). QRT-PCR analysis revealed significant (*P* < 0.05) reduction of *vamp3* (Fig. 7A) or *vamp4* transcripts (Fig. 7B) in *A. phagocytophilum*-infected *vamp3*-dsRNA- or *vamp4*-dsRNA-treated tick cells in comparison to the mock-treated controls, respectively. No differences in the *vamp4* transcripts (Fig. 7C) or *vamp3* transcripts (Fig. 7D) between mock-treated or *vamp3*-dsRNA-treated (Fig. 7C) or *vamp4*-dsRNA-treated (Fig. 7D) tick cells, respectively, were noted. We also noted a significant (*P* < 0.05) reduction in the loads of *A. phagocytophilum* (*p44* DNA levels) in *vamp3*- or *vamp4*-dsRNA-treated tick cells (Fig. 7E) when compared to bacterial loads observed in respective mock-treated controls at 24 hours p.i. QRT-PCR analysis upon combined silencing of both *vamp3* and *vamp4* transcripts revealed significant reduction of bacterial loads in *vamp3 + vamp4*-dsRNA-treated *A. phagocytophilum*-infected tick cells when compared to the levels noted in mock-treated controls (Fig. 7E). Taken together, these results indicate that expressions of VAMP3 and VAMP4 are also important for *A. phagocytophilum* survival at later stages of infection in tick cells.

### Expressions of arthropod *vamp3* and *vamp4* levels are upregulated during acquisition of *A. phagocytophilum* from infected murine hosts into ticks

**Fig 8:**
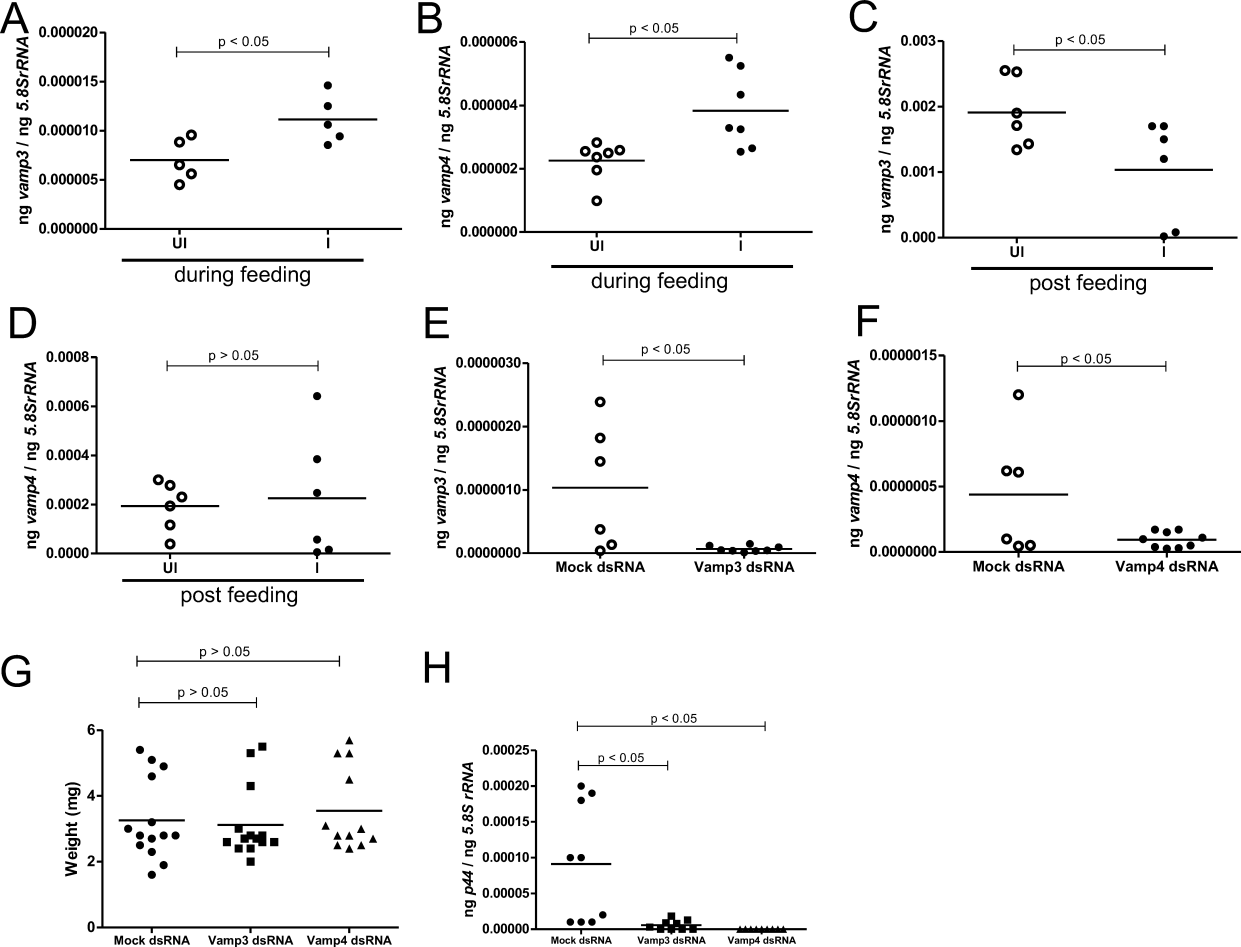
RNAi-mediated knockdown of *vamp3* and *vamp4* expression impairs *A. phagocytophilum* acquisition from infected murine host into ticks. QRT-PCR analysis showing expressions of *vamp3* (**A**) and *vamp4* (**B**) in uninfected unfed during feeding (DF) nymphs that were fed for 48 hours either on uninfected (UI) or *A. phagocytophilum-*infected (**I**) murine hosts. QRT-PCR analysis showing expressions of *vamp3* (**C**) and *vamp4* (**D**) in uninfected unfed nymphs that were repleted (post fed-PF) after being fed on either an uninfected (UI) or *A. phagocytophilum-*infected (**I**) murine hosts. QRT-PCR analysis showing expressions of *vamp3* (**E**) and *vamp4* (**F**) transcripts in fed *A. phagocytophilum-*infected *vamp3*- or *vamp4-*dsRNA-treated ticks, respectively. (**G**) Engorgement weights of the repleted ticks that were either mock-, *vamp3*-, or *vamp4*-dsRNA treated and fed on *A. phagocytophilum-*infected murine hosts are shown. (**H**) *p44* (*A. phagocytophilum* burden) levels in mock-, *vamp3*-, or *vamp4*-dsRNA-treated fed *A. phagocytophilum*-infected ticks are shown. Each data point represents the value from one individual tick. Horizontal lines in the graphs represent the mean of the data points. The mRNA levels of these genes were normalized to *5.8SrRNA* levels. *P* value from Student’s *t*-test is shown. ng on the *Y*-axis indicates nanograms.

Ticks acquire *A. phagocytophilum* via a blood meal upon feeding on an infected vertebrate host. To analyze whether *vamp3* and *vamp4* transcript levels are altered during *A. phagocytophilum* entry into ticks via a blood meal, we performed tick acquisition studies. Uninfected unfed *I. scapularis* nymphs were allowed to feed on *A. phagocytophilum-*infected or uninfected mice. Ticks were either collected at 48 hours from the time they were placed on the mice (during feeding-DF ticks) or were allowed to complete their blood meal and replete (post-feeding-PF ticks). QRT-PCR analysis revealed that the expressions of *vamp3* (Fig. 8A) and *vamp4* (Fig. 8B) were significantly (*P* < 0.05) increased in DF ticks collected from *A. phagocytophilum*-infected mice when compared to levels noted in DF ticks collected from uninfected control mice. Furthermore, we observed a significantly (*P* < 0.05) reduced expression of *vamp3* in PF ticks collected from *A. phagocytophilum*-infected mice when compared to the levels noted in PF ticks repleted from uninfected control mice (Fig. 8C). No significant differences were observed in *vamp4* transcript levels in PF ticks collected from uninfected or *A. phagocytophilum* infected murine hosts (Fig. 8D). These results show that both arthropod *vamp3* and *vamp4* transcripts are upregulated during *A. phagocytophilum* acquisition from infected murine hosts into ticks.

### RNAi-mediated silencing of arthropod *vamp3* and *vamp4* expressions significantly affects *A. phagocytophilum* acquisition from the murine host into ticks

To further investigate the importance of VAMP3 and VAMP4 in *A. phagocytophilum* acquisition from the infected murine host into naïve ticks, we generated *vamp3*- or *vamp4*-deficient unfed uninfected nymphal ticks by RNA interference (RNAi). Unfed uninfected nymphal ticks were either treated with *vamp3*- or *vamp4-* or mock-dsRNAs and allowed to recover for 24 hours. These ticks were then fed on *A. phagocytophilum-*infected murine hosts. QRT-PCR analysis revealed a significant reduction in *vamp3* (Fig. 8E) or *vamp4* (Fig. 8F) transcripts in the *vamp3*-dsRNA or *vamp4*-dsRNA-treated fed ticks, respectively, when compared to the levels noted in mock-dsRNA-treated controls. No differences in the engorgement weights were noted between the mock-, *vamp3*-, or *vamp4*-dsRNA-treated nymphal ticks (Fig. 8G). Comparison of high engorgement weights and low engorgement weights between the groups also revealed no significant difference (Fig. S10). However, we observed a significant (*P* < 0.05) reduction in *A. phagocytophilum* burden (*p44* DNA levels) in *vamp3*-dsRNA- and *vamp4*-dsRNA-treated ticks when compared to the bacterial loads noted in mock-dsRNA-treated controls (Fig. 8H). These results show that VAMP3 and VAMP4 play an important role in the acquisition of *A. phagocytophilum* from the infected murine host into naïve ticks.

### Expressions of arthropod VAMP3 and VAMP4 levels are upregulated in *A. phagocytophilum-*infected unfed nymphal ticks

**Fig 9:**
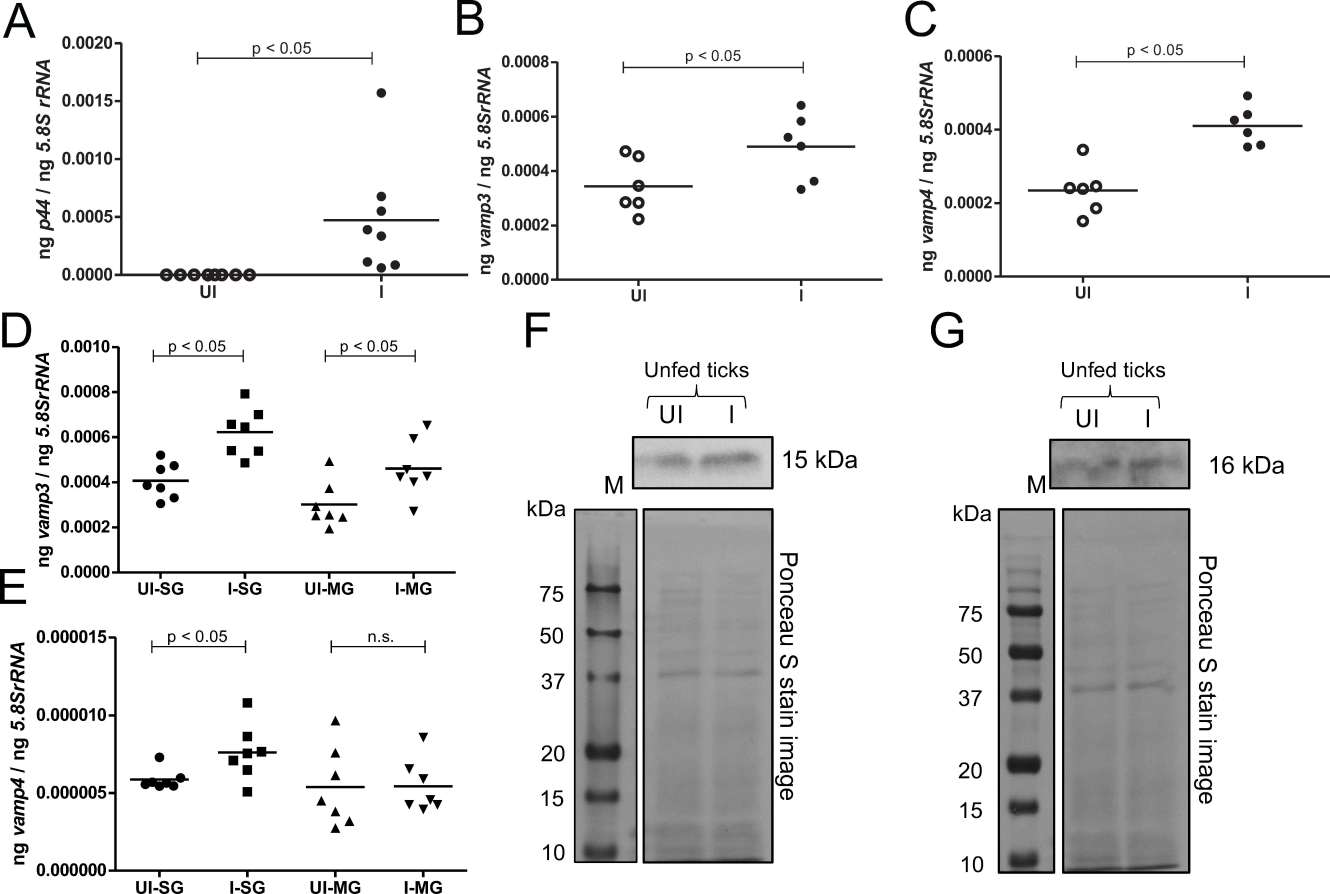
*Anaplasma phagocytophilum* upregulates VAMP3 and VAMP4 transcripts and protein levels in unfed nymphal ticks. QRT-PCR analysis showing *p44* (*A. phagocytophilum* burden) levels (**A**) and expression of *vamp3* (**B, D**) and *vamp4* (**C, E**) transcripts in unfed uninfected (UI) or *A. phagocytophilum-*infected (I) whole nymphs (**A–C**) or salivary glands (SG) and midguts (MG) (**D, E**). Horizontal lines in the graphs represent the mean of the data points. The mRNA levels of these genes and *A. phagocytophilum* p44 levels were normalized to *5.8SrRNA* levels. For data in panels A to C, each data point represents value from one tick, and in panels D and E, each data point represents value from the pool of three SG pairs or MGs collected from three ticks. *P* value from Student’s *t*-test is shown. ng on the *Y*-axis indicates nanograms. Immunoblotting analysis showing VAMP3 (**F**) and VAMP4 (**G**) levels in uninfected (UI) or *A. phagocytophilum-*infected ticks (I). Ponceau S-stained image serves as a loading control image. M indicates protein marker, and molecular mass is shown as kDa.

The data from tick cells show that VAMP3 and VAMP4 are important for both early and later stages of *A. phagocytophilum* infection. We therefore assessed the role of VAMP3 and VAMP4 in *A. phagocytophilum*-infected unfed nymphal ticks. Naïve larvae were fed on *A. phagocytophilum*-infected or uninfected mice and were molted to unfed nymphs. We first determined the bacterial burden in *A. phagocytophilum*-infected molted nymphs. QRT-PCR analysis confirmed the presence of *A. phagocytophilum* in infected unfed nymphs (Fig. 9A). As expected, no *A. phagocytophilum* was detected in uninfected unfed nymphs (Fig. 9A). Furthermore, QRT-PCR analysis showed that both *vamp3* (Fig. 9B) and *vamp4* (Fig. 9C) transcripts were significantly (*P* < 0.05) upregulated in unfed *A. phagocytophilum*-infected nymphs when compared to the levels noted in unfed uninfected ticks. In addition, we noted that *vamp3* (Fig. 9D) and *vamp4* (Fig. 9E) transcripts were significantly (*P* < 0.05) upregulated in salivary glands isolated from unfed *A. phagocytophilum*-infected nymphs when compared to the levels noted in salivary glands isolated from unfed uninfected ticks. In addition, we noted significantly (*P* < 0.05) increased *vamp3* transcripts in midguts isolated from *A. phagocytophilum*-infected unfed nymphs compared to the levels noted in midguts isolated from unfed uninfected nymphs (Fig. 9D). However, no significant (*P* > 0.05) differences in the *vamp4* transcripts were noted in midguts isolated from unfed uninfected or *A. phagocytophilum*-infected nymphs (Fig. 9E). Immunoblotting analysis with anti-VAMP3 and anti-VAMP4 antibodies further supported QRT-PCR results (Fig. 9F and G; Fig. S11) We noted increased VAMP3 and VAMP4 protein levels in unfed *A. phagocytophilum*-infected ticks compared to the levels noted in uninfected controls (Fig. 9F and G; Fig. S11. These results provide evidence that *A. phagocytophilum* upregulates VAMP3 and VAMP4 levels in unfed ticks.

### RNAi-mediated silencing of arthropod *vamp3* and *vamp4* expressions significantly affects *A. phagocytophilum* burden in unfed nymphs.

**Fig 10:**
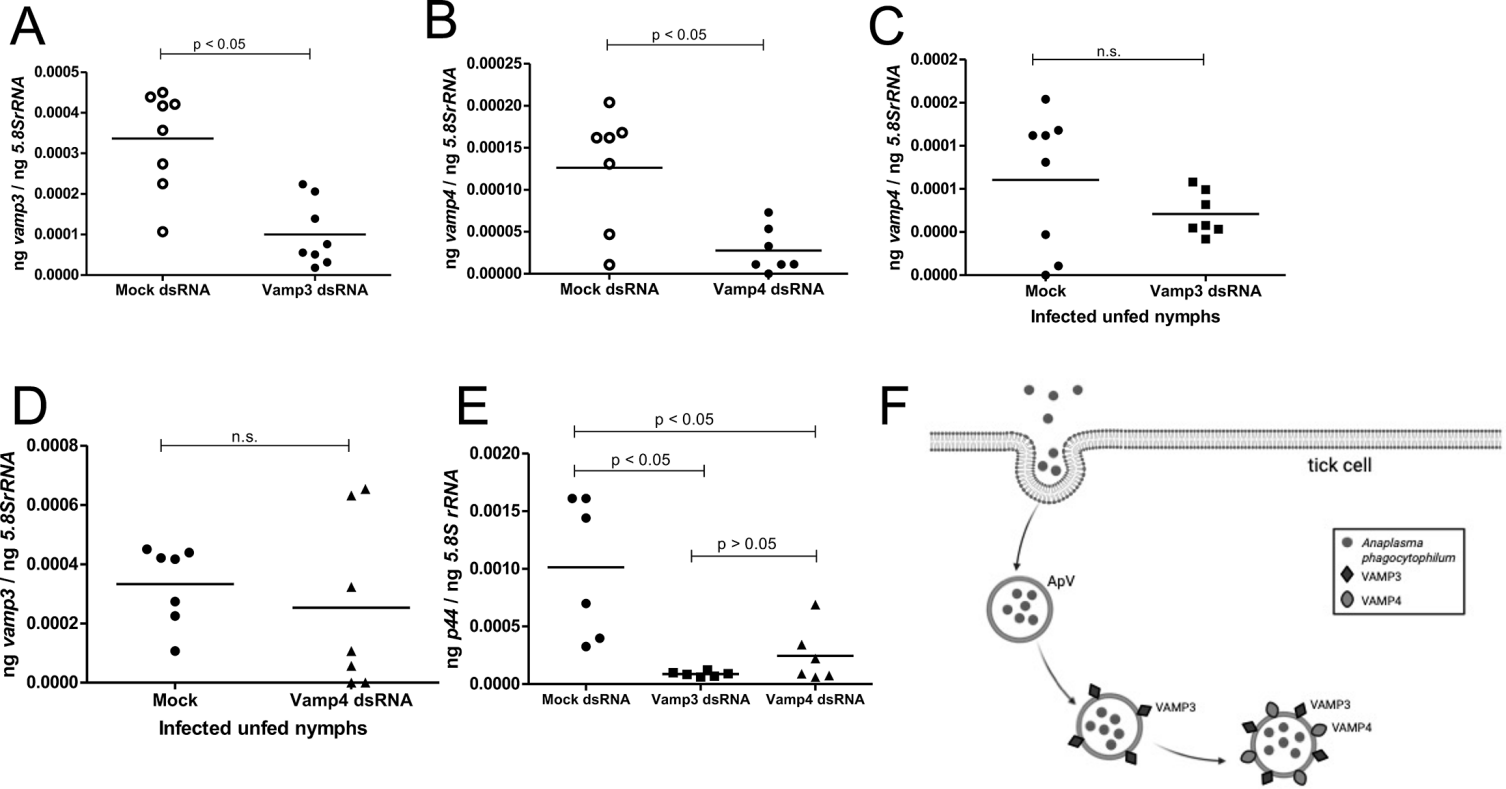
RNAi-mediated silencing of *vamp3* and *vamp4* affects persistent infection of *A. phagocytophilum* in unfed ticks. QRT-PCR analysis showing expression of *vamp3* (**A**) or *vamp4* (**B**) in unfed *A. phagocytophilum-*infected mock, *vamp3*-, or *vamp4-*dsRNA-treated ticks. QRT-PCR analysis showing *vamp4* transcripts in mock or *vamp3*-dsRNA-treated (**C**) or *vamp3* transcripts in mock or *vamp4*-dsRNA-treated (**D**) in unfed *A. phagocytophilum*-infected nymphs. (**E**) *p44* (*A. phagocytophilum* burden) levels in mock-, *vamp3*-dsRNA-, or *vamp4*-dsRNA-treated unfed *A. phagocytophilum-*infected ticks are shown. Horizontal lines in the graphs represent the mean of the data points. The mRNA levels of these genes were normalized to *5.8SrRNA* levels. *P* value from Student’s *t*-test is shown. ng on the *Y*-axis indicates nanograms. (**F**) Model showing the role of VAMP3 and VAMP4 in *A. phagocytophilum* infection in tick cells. An endocytic cup is formed (with membrane invagination) for uptake of *A. phagocytophilum*. Upon entry, *A. phagocytophilum* further forms a host-membrane-derived vacuole called morulae that has localization of VAMP3 and VAMP4 on the surface of this pathogen-containing vacuole (ApV). Based on the results, we believe that VAMP3 and VAMP4 are important for both early and persistent infection of *A. phagocytophilum* in tick cells. The picture is not drawn to the scale. This image was created with BioRender.com.

To further analyze the importance of VAMP3 and VAMP4 during the persistent infection of *A. phagocytophilum* in ticks, we generated *vamp3*- or *vamp4*-deficient unfed *A. phagocytophilum-*infected nymphal ticks by RNA interference (RNAi). Unfed *A. phagocytophilum-*infected nymphal ticks were either treated with *vamp3*- or *vamp4-* or mock-dsRNA and allowed to recover for 24 hours. QRT-PCR analysis revealed a significant (*P* < 0.05) reduction in *vamp3* (Fig. 10A) and *vamp4* (Fig. 10B) mRNA in the *vamp3*-dsRNA- and *vamp4*-dsRNA-treated ticks, respectively, when compared to the levels noted in the mock-dsRNA-treated controls. We noted no differences in the *vamp4* transcripts (Fig. 10C) or *vamp3* transcripts (Fig. 10D) between mock-treated or *vamp3*-dsRNA-treated (Fig. 10C) or *vamp4*-dsRNA-treated (Fig. 10D) ticks. However, we noted a significant (*P* < 0.05) reduction in the loads of *A. phagocytophilum* in *vamp3*-dsRNA- or *vamp4*-dsRNA-treated ticks (Fig. 10E) when compared to the bacterial burden observed in mock-dsRNA-treated controls. Collectively, the combined results from *in vitro* and *in vivo* infection models show that the arthropod VAMP3 and VAMP4 are not only important in the early and at later phases of *A. phagocytophilum* infection of tick cells but are also important for bacterial acquisition from an infected vertebrate host to ticks.

## DISCUSSION

In mammalian cells, *A. phagocytophilum*-containing vacuoles are decorated with SNARE proteins of the trans-Golgi network such as VAMP4 and syntaxin 16 (35). The molecular players important in *A. phagocytophilum* entry and in the formation of morulae in tick cells are poorly understood. In this study, we provided evidence that arthropod VAMP3 and VAMP4 proteins play an important role not only in the early phase of *A. phagocytophilum* infection in tick cells but also in the persistent survival of this bacterium at the later stages of infection in tick cells and ticks (Fig. 10F).

A study on the invasion and morphogenesis of the human granulocytic ehrlichiosis (HGE) agent in tick cells showed that these bacteria were found attached to tick cells within the first hour of infection (42). By 4 hours post-infection, tick cells were seen to engulf the bacterium by a process similar to phagocytosis (42). By 48 hours post-infection, the replication of this bacteria was well-established in tick cells (42). Post 48 hours, these bacteria were found to be exiting from infected cells and invading uninfected neighboring cells to continue the infection (42). In our study, significantly increased *vamp3* and *vamp4* transcripts at 4 hours post-infection indicate an important role for both these proteins in the early phase of *A. phagocytophilum* infection. This observation was further supported by results of genetic knockdown/silencing studies, followed by those of microscopic and QRT-PCR analysis. In both these independent analyses, we noted similar observations of significantly reduced *A. phagocytophilum* burden upon silencing of *vamp3* and *vamp4,* either individually or in combination at 4 hours post-infection. Collectively, our study findings support previous observations and indicate that *A. phagocytophilum* enters tick cells within 4 hours and VAMP3 and VAMP4 are important for this early phase of infection.

Mammalian SNARE proteins like VAMPs are known to be exploited by various intravacuolar bacteria (43–45). *Salmonella* sp. induces phagocytotic entry into the host cells by using VAMP8 at the entry site (43); recruits VAMP2, VAMP3, and VAMP4 on to the *Salmonella*-containing vacuoles during infection (44, 46); and uses VAMP7 for secretion of typhoid toxins outside the host cell (44). *Chlamydia* sp. recruits host VAMP3 and VAMP4 to aid development of chlamydial inclusion and lipid acquisition (33, 47, 48). Another intracellular pathogen, *Coxiella burnetii*, uses VAMP3, VAMP7, and VAMP8 for the development of *Coxiella*-containing vacuoles (45). In this study, we provide evidence for another obligate intracellular bacteria (*A. phagocytophilum*) to this list that exploits VAMP3 and VAMP4 proteins for its entry and survival in its vector host. Based on the findings from our study and those of others, we conclude that SNAREs-VAMPS are the common conserved molecules exploited by intracellular bacterial pathogens for their niche and survival in the mammalian and vector hosts.

We noted that tick VAMP3 and VAMP4 have several post-translational modification sites. Studies from mammalian cells have also reported that VAMP3 and VAMP4 indeed get phosphorylated (49, 50). Both VAMP3 and VAMP4 have multiple roles in exocytic and endocytic pathways (51, 52). Therefore, phosphorylation of VAMP3 and VAMP4 could be important for their roles in entry/exit pathways. The significance of the presence of these phosphorylation sites on tick VAMP3 and VAMP4 is currently not known. However, we believe that phosphorylation on tick VAMP3 and VAMP4 may direct these proteins for similar roles in tick cells. In addition, we noted that both *vamp3* and *vamp4* transcripts are expressed at all developmental stages of ticks. Significantly increased transcripts of *vamp3* transcripts in female adult ticks compared to nymphal ticks suggest a role for this protein in female tick biology. A study has suggested that SNARE proteins are important for tick blood feeding and oviposition in *Amblyomma* ticks (53). Future studies would unravel whether VAMP3 has a similar role in the oviposition in female *I. scapularis* ticks.

*Anaplasma phagocytophilum* enters into ticks via a blood meal (13). It first enters the midgut, crosses the midgut epithelium barrier, infects hemocytes, and then colonizes in tick salivary glands (13, 54). By 48 hours during tick feeding, *A. phagocytophilum* can be easily detectable (by PCR) in the salivary glands (54). In our study, the observation of significantly increased *vamp3* and *vamp4* transcripts in 48 hours during feeding ticks and in tick salivary glands strongly supports that these molecules are important for bacterial acquisition from an infected vertebrate host into ticks and in colonization within ticks. The downregulation of *vamp3* transcripts, but not *vamp4,* in post-feeding ticks suggests a temporal role for these molecules during feeding. We believe that initial upregulation of VAMP3 and VAMP4 at 48 hours during feeding is important and sufficient for *A. phagocytophilum* acquisition into ticks. Soon after entry, *A. phagocytophilum* starts initial replication to colonize ticks (54). VAMP3 is also involved in several membrane trafficking events including immune signaling events (51, 55). Therefore, bacterial responses to downregulate this protein to inactivate tick immune signaling cannot be ruled out. Even though these molecules may play a temporal role during tick feeding, the data from RNAi studies strongly support that they are important for the bacterial acquisition from infected vertebrate hosts into ticks. The data noted from microscopic studies indicate that VAMP3 and VAMP4 efficiently localize to *A. phagocytophilum*-containing vacuoles in persistently infected tick cells. In addition, the significant reduction of *A. phagocytophilum* burden upon silencing of *vamp3* and *vamp4* transcripts in unfed nymphal ticks or in tick cells after 24 hours p.i. clearly indicates that VAMP3 and VAMP4 are required for persistent survival of this bacterium in both tick cells and ticks.

In summary, our study indicates that VAMP3 and VAMP4 are not only important for the early and late phase of *A. phagocytophilum* infection in tick cells and ticks but also are important for the acquisition of this bacterium from an infected host into ticks. Understanding biology of tick*–A. phagocytophilum* interactions could lead to the development of strategies to target survival of this bacterium in ticks.

## MATERIALS AND METHODS

### Bacteria

*Anaplasma phagocytophilum* isolate NCH-1 (obtained from BEI Resources, NIAID, NIH), or *A. phagocytophilum* carrying GFP cassette or mCherry cassette referred as *A. phagocytophilum or GFP-A. phagocytophilum* or mCherry-*A. phagocytophilum* was used throughout this study. *Anaplasma phagocytophilum* or *GFP-A. phagocytophilum* was maintained in HL-60 cells and isolated from these cells (24, 26, 56). Briefly, infected HL-60 cells were centrifuged for 10 minutes at 2,950 × *g* at 4°C, and the pellet was washed two times with sterile 1× phosphate-buffered saline (PBS). Cells were then re-suspended in 1× IMDM medium and processed for a freeze/thaw cycle, followed by passing through 25- and 27-gauge needles six to eight times. The obtained suspension was later centrifuged at 260 × *g* for 3 minutes to generate host cell-free bacteria in the supernatant. This supernatant was used for infection of tick cells in this study.

### Ticks

*Ixodes scapularis* ticks (larvae, nymphs, and adult male and females) used in this study were obtained from BEI Resources (CDC), NIAID, NIH. Tick rearing was conducted in an Environmental Chamber from Parameter Generation and Control, USA. The incubator was set at 23 ± 2°C with 94% relative humidity and 14:10 light:dark conditions.

### Tick cell line

The *I. scapularis* tick cell line, ISE6, purchased from ATCC, was grown in L15B300 medium prepared from Leibovitz’s L-15 medium, powder with 5% tryptose phosphate broth, 5% heat-inactivated FBS, and 0.1% bovine lipoprotein concentrate, pH 7.2. ISE6 cell cultures were maintained at 34°C (18, 24, 26). GFP*–Anaplasma phagocytophilum*-infected tick cells were obtained from Dr. Munderloh (University of Minnesota, MN, USA) and maintained like an uninfected ISE6 cell line.

### Mice, tick feeding, and acquisition studies

C57BL/6J and B6.129S7-Rag1tm1Mom/J (RAG^−/−-^) (female, 4–6 weeks, Jackson Laboratories, USA) mice were used in this study. *Anaplasma phagocytophilum* infection was maintained in B6.129S7-Rag1tm1Mom/J (RAG^−/−^) mice. Uninfected larval ticks were fed on either uninfected or *A. phagocytophilum*-infected mice and were molted to nymphs. Unfed uninfected or *A. phagocytophilum-*infected nymphal ticks were processed for DNA or RNA extractions and were used for QRT-PCR analysis. For acquisition studies, unfed uninfected nymphs were fed on uninfected or *A. phagocytophilum*-infected C57BL/6 J mice. During feeding (DF), ticks were collected at 48 hours post-tick placement onto the mice, and repleted post-fed (PF) ticks were collected at 72–96 hours post-tick placement onto the mice. For VAMP RNAi-mediated silencing studies, unfed *A. phagocytophilum*-infected nymphs were bathed in 200 µL 1 x PBS solution containing 1 µg of *vamp3-*, or *vamp4-,* or mock-dsRNA for 40 minutes at 34°C. Ticks were washed three times with 1 x PBS and stored in the tick environmental chamber for 24 hours. These ticks were then fed on naïve uninfected C57BL/6 J mice. Engorged ticks were collected after repletion, weighed, and processed further for DNA and RNA extractions, followed by QRT-PCR analysis to measure the bacterial loads and silencing efficiency.

### Total RNA and DNA isolation and QRT-PCR analysis

The Aurum Total RNA Mini kit (BioRad, USA) was used to extract total RNA from ticks and tick cells (2 × 10^5^) (22, 25, 56). BioRad iScript cDNA synthesis kit (BioRad, USA) was used to generate cDNA (22, 25, 56). The cDNA was used as a template for amplifying *vamp3*, *vamp4*, *vamp7,* and housekeeping gene (*5.8S rRNA*) fragments. DNA from ticks and tick cells were extracted using the DNeasy blood and tissue kit (Qiagen, USA). The oligonucleotide sequences for *5.8S* rRNA are mentioned in our previous publications (23, 56). The oligonucleotides used in this study are shown in Table S1. QRT-PCR was performed using MAXIMA SYBR/R Green (ThermoScientific, USA) and CFX96 Touch System (BioRad, USA) (22,25,56). The *5.8S rRNA* amplicons were quantified as an internal control and to normalize the amount of the template. The standard curves for each gene fragment were prepared with tenfold serial dilutions starting from 1 to 0.000001 ng/μL of known quantities of respective gene fragments.

### dsRNA synthesis

The dsRNA synthesis was performed as previously described (24, 57, 58). Briefly, *vamp3- or vamp4-*dsRNA fragments were generated by PCR using gene-specific primers containing BglII (forward primer) and KpnI (reverse primer) restriction enzyme sites using oligonucleotides mentioned in Table S1. The fragments containing *vamp3* or *vamp4* sequences were purified and cloned into BglII-KpnI sites of the L4440 double T7 Script II vector. The dsRNAs complementary to *vamp3* or *vamp4* gene sequences were synthesized using the MEGAscript RNAi Kit (Ambion Inc.) and by following the manufacturer’s instructions. Mock dsRNA was prepared from the multiple cloning site region of the empty pL4440 vector.

### Tick cell line experiments

Tick cells (2 × 10^5^) were seeded onto 12-well plates and incubated for 16–20 hours. Following incubation, tick cells were infected with *A. phagocytophilum* (isolated from 4 × 10^5^ NCH-1-infected HL-60 cells/well). Tick cells were then incubated and harvested at 4 hours, 8 hours, 24 hours, 48 hours, and 72 hours post-infection. The samples were processed for RNA extraction to measure *vamp3, vamp4,* and *vamp7* transcripts.

### Tick cell line experiments with dsRNA treatment

Tick cells (2 × 10^5^) were seeded onto 12-well plates and incubated for 16–20 hours. Following incubation, tick cells were transfected with 750 ng of mock-, *vamp3*-, or *vamp4*-dsRNA for 24 hours, followed by *A. phagocytophilum* (isolated from 4 × 10^5^ NCH-1-infected HL-60 cells or 4 × 10^5^ GFP-*A. phagocytophilum-*infected tick cells/well) infection. The cells were then incubated, harvested at 4 hours and 24 hours post-infection, and processed further for RNA and DNA extractions to measure *vamp3* and *vamp4* transcripts for silencing efficiency and the bacterial loads, respectively.

### Immunofluorescence and confocal microscopy

For colocalization studies, GFP-*A. phagocytophilum-*infected tick cells (2 × 10^5^) were seeded onto 12-well plates with glass coverslips and incubated for 16–20 hours. Cells were washed in PBS, fixed in 4% paraformaldehyde for 10 minutes at room temperature, permeabilized with 0.2% Triton X-100 for 10 minutes at room temperature, and blocked with 3% bovine serum albumin in 1XPBS for 30 minutes at 37°C. Primary antibodies directed against either VAMP3 or VAMP4 (ABclonal, USA) were used at a dilution of 1:250 in 1% BSA in 1x PBS. Goat anti-rabbit secondary antibody conjugated with Alexa Fluor 594 (Molecular probes/ThermoScientific, USA) was used at a dilution of 1:2,500 in 1% BSA in 1 x PBS. Cells were counterstained for nuclei with DAPI (1 µg/mL) for 2 minutes at room temperature. The coverslips were mounted onto glass slides, and images were captured using a Leica SP8 laser-scanning confocal microscope (Leica Microsystems, Wetzlar, Germany). All the images were captured by using a 63 x oil immersion lens. Images were captured by sequential scanning, where DAPI was excited by 405-nm UV laser (emission 415–470 nm), GFP was excited by 488-nm laser (emission 494–550 nm), and Alexa 594 was excited by using 594-nm laser (emission 601–670 nm). For confocal microscopy of dsRNA-treated cells, uninfected tick cells (2 × 10^5^) were seeded onto 12-well plates with glass coverslips and incubated for 16–20 hours. Following incubation, tick cells were transfected with 750 ng of mock- or *vamp3*- or *vamp4*- dsRNA for 24 hours, followed by *A. phagocytophilum* (isolated from 4 × 10^5^ GFP-*A. phagocytophilum-*infected tick cells) infection. Four hours post-infection, cells were fixed, permeabilized, blocked, and stained with respective antibodies and processed for microscopy as mentioned earlier.

### Microscopic quantification of VAMP3- or VAMP4-positive *A. phagocytophilum*-occupied morulae

Uninfected tick cells (2 × 10^5^) were seeded onto 24-well plates with glass coverslips and incubated for 16–20 hours. Following incubation, tick cells were infected with mcherry-*A. phagocytophilum* (isolated from 4 × 10^5^ mcherry-*A. phagocytophilum-*infected HL60 cells). At 4 hours, 24 hours, and 48 hours post-infection, cells were washed with 1X PBS, fixed with 4% paraformaldehyde for 10 minutes at room temperature, permeabilized with 0.2% Triton X-100 for 10 minutes at room temperature, and blocked with 3% bovine serum albumin in 1× PBS for 30 minutes at 37°C. Primary antibodies directed against VAMP3 or VAMP4 (ABclonal, USA) were used at a dilution of 1:250 in 1% BSA in 1× PBS. Goat anti-rabbit secondary antibody conjugated with Alexa Fluor 488 (Molecular probes/ThermoScientific, USA) was used at a dilution of 1:2,500 in 1% BSA in 1× PBS. Cells were counterstained for nuclei with DAPI (1 µg/mL) for 2 minutes at room temperature. The coverslips were mounted onto glass slides, and images were captured using a laser-scanning confocal microscope (LSM 510; Carl Zeiss, Inc.). Number of VAMP3- or VAMP-4 positive morulae were counted from 10 images for each time point and used for plotting the graph.

### Tick experiments with dsRNA

Uninfected unfed *I. scapularis* nymphs were bathed in 1 µg mock- or *vamp3-* or *vamp4*-dsRNA in 200 μL 1 × PBS for 40 minutes at 34°C. Ticks were washed three times with 1x PBS and stored in the tick environmental chamber for recovery for 24 hours. Recovered dsRNA-treated nymphs were then used for acquisition studies on mice, as mentioned earlier.

### Sequence alignment and bioinformatic analysis

GenBank accession numbers for the nucleotide and amino acid sequences used in this study are mentioned in Table S2 and S3. Amino acid sequence alignments for VAMP3 and VAMP4 orthologs from various organisms were performed using DNASTAR CLUSTALW alignment software. Sequence analyses were performed for *I. scapularis* VAMP3 and VAMP4 with various organisms. The phylogenetic tree was constructed using the neighbor-joining method (BIONJ) and BIONJ algorithm in DNSTAR. The amino acid sequences for *I. scapularis* VAMP3 and VAMP4 proteins were downloaded from GenBank and individually analyzed at PROSITE (http://prosite.expasy.org/) for the prediction of myristoylation, protein kinase C phosphorylation, casein kinase II phosphorylation, and ATP/GTP binding sites and as described previously (59,60)

### Immunoblotting

Ten uninfected or *A. phagocytophilum*-infected unfed nymphs were crushed and homogenized using a pellet pestle cordless motor (Biospec, OK) and pellet pestle (VWR, USA) in modified-RIPA lysis buffer, supplemented with EDTA-free protease inhibitor cocktail to generate tick lysates from uninfected or infected ticks. Protein concentrations were determined by the Bradford (BCA) protein assay kit and as per the manufacturer’s recommendations. Tick lysates (30 mg) were mixed with Laemmli sample buffer, boiled for 5 minutes, and resolved on 12% reducing SDS-PAGE gels. Gels were run at 110 V. Gels were later transferred to nitrocellulose membranes. Post-transfer, the membrane was stained with PonceauS Stain. PonceauS-stained membrane images served as loading controls. Membrane sections were blocked with 5% BSA in 1× TBST (1× TBS, 0.05% Tween 20). Primary antibodies directed against VAMP3 and VAMP4 (ABclonal, USA) were used at a dilution of 1:1,000 in 5% BSA in 1× TBST. HRP-conjugated goat anti-rabbit secondary antibody was used at a dilution of 1:5,000 in 5% BSA in 1× TBST. Development of the chemiluminescent substrate was visualized using a BioRad ChemiDoc Touch Imaging System (BioRad, USA).

### Quantification and statistical analysis

Statistical significance in the data sets was analyzed using GraphPad Prism6 software (https://www.graphpad.com/) and Microsoft Excel 2010 (https://www.microsoft.com). Student’s t-test was used to compare the statistical significance between the groups, or ANOVA was used to compare the multiple group’s variations. *P* values of < 0.05 were considered significant in all analyses.

## SUPPLEMENTAL MATERIAL

Supplemental material mbio.03549-24-s0001.pdf Supplemental figures, tables, and video legends.

Video S1 mbio.03549-24-s0002.mp4 Z stack video showing localization of arthropod VAMP3 around *A. phagocytophilum* in tick cells.

Video S2 mbio.03549-24-s0003.mp4 3D imaging showing localization of arthropod VAMP3 around *A. phagocytophilum* in tick cells.

Video S3 mbio.03549-24-s0004.mp4 Z stack video showing localization of arthropod VAMP4 around *A. phagocytophilum* in tick cells.

Video S4 mbio.03549-24-s0005.mp4 3D imaging showing localization of arthropod VAMP4 around *A. phagocytophilum*in tick cells.

ASM does not own the copyrights to Supplemental Material that may be linked to, or accessed through, an article. The authors have granted ASM a non-exclusive, world-wide license to publish the Supplemental Material files. Please contact the corresponding author directly for reuse.
