# Supplementary material for: Rickettsial pathogen augments tick vesicular-associated membrane proteins for infection and survival in the vector host
Source: mBio. 2025 Feb 14;16(3):e03549-24. doi: 10.1128/mbio.03549-24 (PMC11898744; doi:10.1128/mbio.03549-24)
Supplement: Supplemental material — Supplemental figures, tables, and video legends. [file mbio.03549-24-s0001.pdf]

Supplementary Figure legends

Supplementary Figure 1. *vamp2, vamp7* and *vamp33* levels are not modulated in the early time points of *A. phagocytophilum* infection in tick cells. Quantitative PCR analysis showing expression of *vamp2* (**A**), *vamp7* **(B)** and *vamp33* (**C**) in uninfected (UI) or *A. phagocytophilum*-infected (I) tick cells. Each circle represents data from one independent culture plate well. Horizontal lines in the graphs represent mean of the data points. The mRNA levels of these genes were normalized to *5.8SrRNA* levels. P value from Student's t-test is shown.

Supplementary Figure 2. *I*xodes *scapularis* VAMP3 (**A**) and VAMP4 (**B**) amino acid sequence alignment with other orthologs using ClustalW program in DNASTAR is shown. Residues that match are shaded in black color. GenBank accession numbers for the *I. scapularis* (Is), *Homo sapiens* (Hs), *Mus musculus* (Mm), *Drosophila melanogaster* (Dm), *Anopheles gambiae* (Ag), *Culex quinquifasciatus* (Cq) and *Aedes aegypti* (Aa) sequences are shown on the left side.

Supplementary Figure 3. Sequence similarity and identity between *I. scapularis* VAMP3 and VAMP4 proteins with respective orthologs from other organisms. The percent identity (horizontally above black boxed diagonal line) and percent similarity (vertically below black boxed diagonal line) of the *I. scapularis* VAMP3 (**A**) and VAMP4 (**B**) amino acid sequence in comparison to the ortholog proteins from *Homo sapiens* (Hs), *Mus musculus* (Mm), *Drosophila melanogaster* (Dm), *Anopheles gambiae* (Ag), *Culex quinquifasciatus* (Cq) and *Aedes aegypti* (Aa) are shown. Sequence similarity and identity data were generated based on the CLUSTALW

alignment of the sequences in DNASTAR. GenBank accession numbers for the sequences are mentioned along with the organism names.

Supplementary Figure 4. Phylogenetic analysis of *I. scapularis* VAMP3 (A) and VAMP4 (B) amino acid sequences with other orthologs is shown. The phylogenetic tree was generated in DNASTAR using the Neighbor-Joining (BIONJ) method with BIONJ algorithm.

Supplementary Figure 5. Sequence analysis of *I. scapularis* VAMP3 and VAMP4 with orthologs from other ticks. Pie-chart represents amino acid percent identity of *I. scapularis* VAMP3 (**A**) and VAMP4 (**B**) amino acid sequences with ortholog proteins from *Amblyomma americanum* (Ama), *Dermacentor andersoni* (Da), *Ixodes hexagonus* (Ih) and *Rhipicephalus sanguineus* (Rs). The percent identity (horizontally above black boxed diagonal line) and percent similarity (vertically below black boxed diagonal line) of the *I. scapularis* VAMP3 (**C**) and VAMP4 (**D**) amino acid sequence in comparison to the ortholog proteins from other ticks are shown. Sequence similarity and identity data was generated based on the CLUSTALW alignment of the sequences in DNASTAR. GenBank accession numbers for the sequences are mentioned along with the organism names.

Supplementary Figure 6. Amino acid sequence alignment and phylogenetic analysis of I. scapularis VAMP3 and VAMP4 with orthologs from other ticks. *I. scapularis* VAMP3 (**A**) and VAMP4 (**B**) amino acid sequence alignment with other tick orthologs using ClustalW program in DNASTAR is shown. Residues that match are shaded in black color. GenBank accession numbers for the *I. scapularis* (Is), *Amblyomma americanum* (Ama), *Dermacentor*

andersoni (Da)*, Ixodes hexagonus* (Ih) and *Rhipicephalus sanguineus* (Rs) sequences are shown on the left side. The phylogenetic tree for VAMP3 **(C)** and VAMP4 **(D)** was generated in DNASTAR using the Neighbor-Joining (BIONJ) method with BIONJ algorithm.

Supplementary Figure 7. *vamp3*- or *vamp4*-dsRNA treatment showed no morphological changes in tick cells at early time point of infection (4 h p.i.). Representative images of mock-treated, *vamp3*-dsRNA treated, *vamp4*-dsRNA treated or *vamp3*+*vamp4*-dsRNA treated at pre-treatment (untreated), 24 h post dsRNA transfection, and 28 h post-transfection and 4 h post-infection is shown. All groups were infected with *A. phagocytophilum*. Scale bar indicates 200 μm.

Supplementary Figure 8. RNAi-mediated silencing of *vamp3* and *vamp4* affects expression of VAMP3 and VAMP4 protein, respectively, and early phase of *A. phagocytophilum* infection in tick cells. Confocal microscopic images of GFP-*A. phagocytophilum* (green)-infected tick cells at 4 h p.i. probed with anti-VAMP3 (**A**) or -VAMP4 (**B**) antibodies, followed by the incubation with Alexa Flour 594-conjugated secondary antibody (red). Cells were also stained with DAPI to visualize the nuclei (blue). Original magnification of 63x image is shown. Scale bar represents 5 μm.

Supplementary Figure 9. *vamp3*- or *vamp4*-dsRNA treatment showed no morphological changes in tick cells at later time point of infection (24 p.i.). Representative images of *A. phagocytophilum*-infected mock-treated, *A. phagocytophilum*-infected *vamp3*-dsRNA treated, *A. phagocytophilum*-infected *vamp4*-dsRNA treated or *A. phagocytophilum*-infected

vamp3+*vamp4*-dsRNA treated at pre-treatment (untreated), 24 h post dsRNA transfection, and 48 h post-transfection and 24 h post-infection is shown. Scale bar indicates 200 µm.

Supplementary Figure 10. RNAi-mediated knockdown of *vamp3* or *vamp4* expression in ticks has no effect on engorgement. Engorgement weights of the repleted ticks that were either mock-, *vamp3*- or *vamp4*-dsRNA treated and fed on *A. phagocytophilum*-infected murine hosts are shown. The data shown in figure 8G was split into lighter (**A**) or heavier (**B**) groups and then plotted separately. Horizontal lines in the graphs represent mean of the data points. P value from Student's t-test is shown. Each data point represents an individual tick.

Supplementary Figure 11. Immunoblotting analysis of VAMP3 and VAMP4 in ticks. Full length immunoblot images for the cropped images for VAMP3 and VAMP4 in Figure nine is shown. Immunoblotting analysis showing VAMP3 and VAMP4 levels in uninfected (UI) or *A. phagocytophilum* infected ticks (I). Ponceau S-stained image serves as a loading control image. M indicates protein marker and molecular mass is shown as kDa.

Supplementary Table 1. Oligonucleotides used in this study. List of all oligonucleotides used in this study are shown. The sequences are from 5' to 3'.

Supplementary Table 2. GenBank nucleotide accession numbers. The accession numbers for *I. scapularis vamp* genes are shown.

Supplementary Table 3. GenBank accession numbers for proteins analyzed in this study. The accession numbers for proteins analyzed in this study are shown.

Supplementary Video 1. Z-stack video showing VAMP3 localization. Z-stack video of fixed cells showing the colocalization of *A. phagocytophilum*-containing vacuoles with VAMP3 distribution in different focal planes. Red: VAMP3 staining, Green: GFP-*A. phagocytophilum*, Blue-DAPI staining.

Supplementary Video 2. 3D-imaging video showing VAMP3 localization. 3D-imaging video of fixed cells showing the colocalization of *A. phagocytophilum*-containing vacuoles with VAMP3 distribution in different focal planes. Red: VAMP3 staining, Green: GFP-*A. phagocytophilum*, Blue-DAPI staining.

Supplementary Video 3. Z-stack video showing VAMP4 localization. Z-stack video of fixed cells showing the colocalization of *A. phagocytophilum*-containing vacuoles with VAMP4 distribution in different focal planes. Red: VAMP4 staining, Green: GFP-*A. phagocytophilum*, Blue-DAPI staining.

Supplementary Video 4. 3D-imaging video showing VAMP4 localization. 3D-imaging video of fixed cells showing the colocalization of *A. phagocytophilum*-containing vacuoles with VAMP4 distribution in different focal planes. Red: VAMP4 staining, Green: GFP-*A. phagocytophilum*, Blue-DAPI staining.

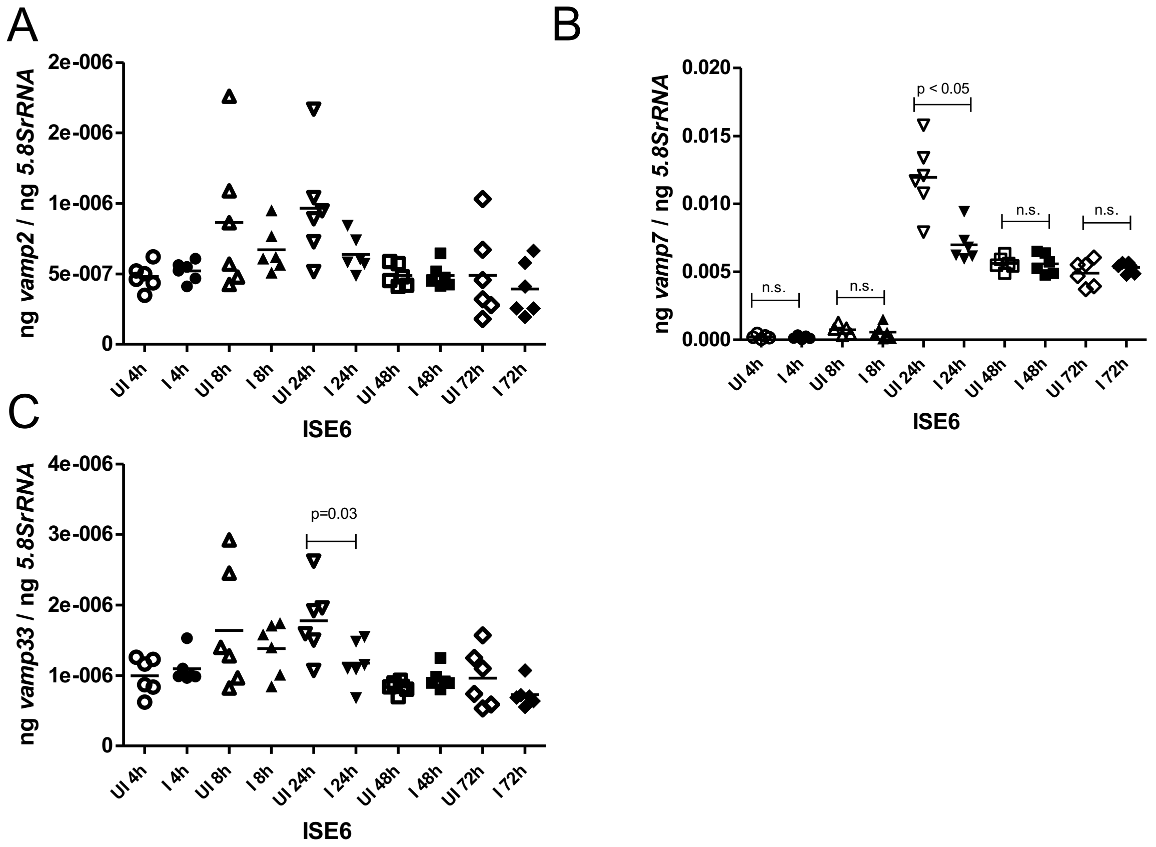

Supplementary Figure 1

A

B

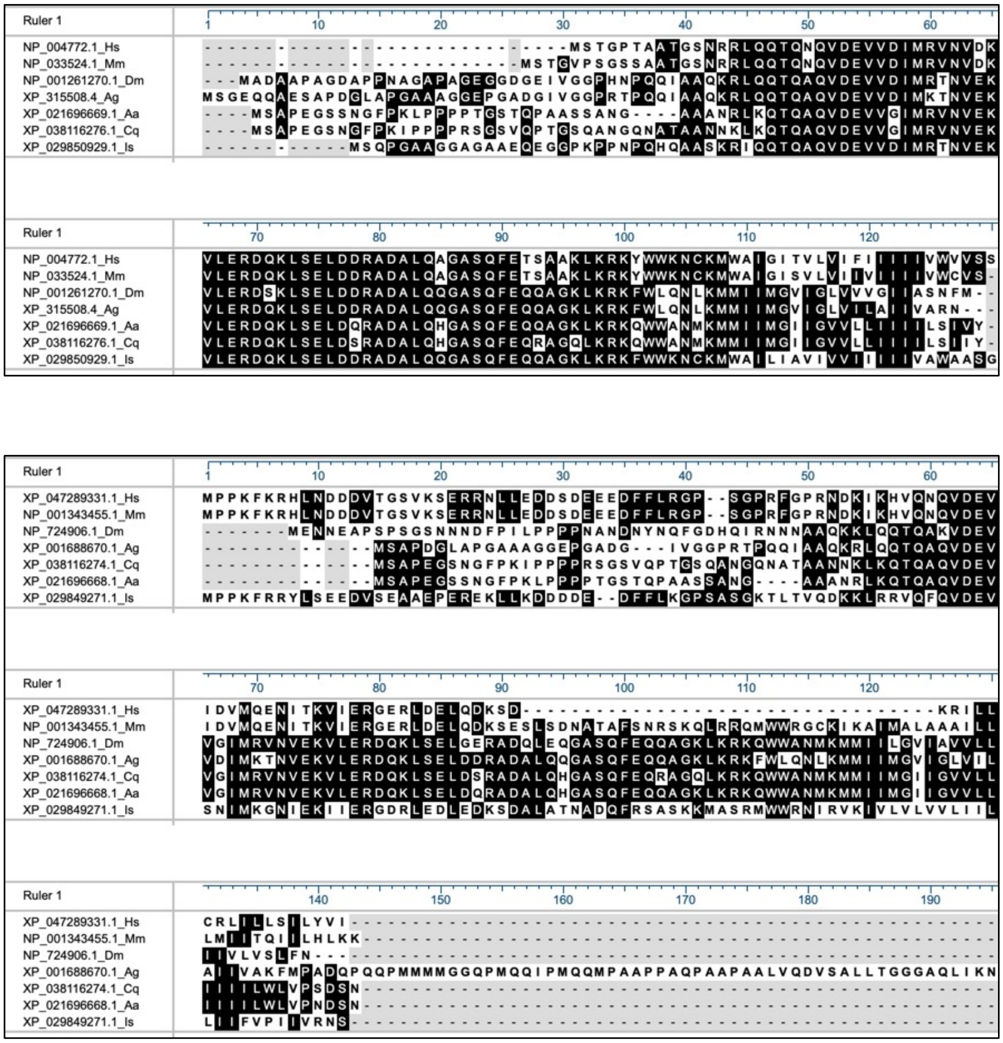

VAMP3

VAMP4

Supplementary Figure 2

A

Vamp3			% Identity						
			A	B	C	D	E	F	G
% Similarity	A	NP_004772.1_Hs		91.4	60.22	60.22	64.52	62.37	72.04
	B	NP_033524.1_Mm	95.7		61.29	61.29	63.44	62.37	72.04
	C	NP_001261270.1_Dm	69.89	72.04		88.17	72.04	68.82	74.19
	D	XP_315508.4_Ag	72.04	74.19	92.47		73.12	69.89	76.34
	E	XP_021696669.1_Aa	73.12	73.12	80.65	80.65		93.55	68.82
	F	XP_038116276.1_Cq	73.12	74.19	80.65	80.65	96.77		65.59
	G	XP_029850929.1_Is	81.72	84.95	82.8	83.87	77.42	78.49	

B

Vamp4			% Identity						
			A	B	C	D	E	F	G
% Similarity	A	XP_047289331.1_Hs		88.75	25	23.75	31.25	27.5	48.75
	B	NP_001343455.1_Mm	91.25		25	23.75	30	26.25	51.25
	C	NP_724906.1_Dm	51.25	55		50	62.5	56.25	28.75
	D	XP_001688670.1_Ag	46.25	48.75	62.5		57.5	60	32.5
	E	XP_038116274.1_Cq	52.5	55	72.5	67.5		83.75	31.25
	F	XP_021696668.1_Aa	51.25	53.75	70	70	86.25		30
	G	XP_029849271.1_Is	75	77.5	56.25	52.5	56.25	55	

Supplementary Figure 3

A VAMP3

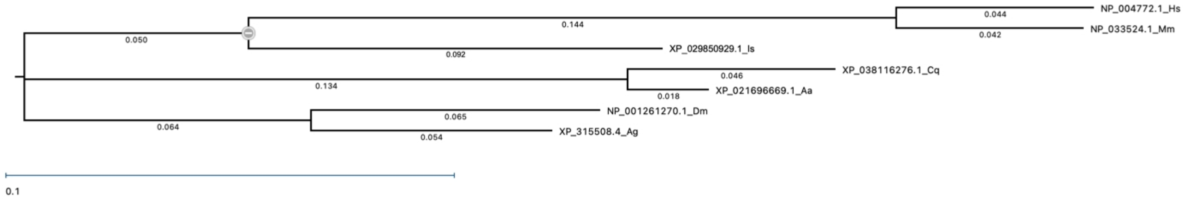

B VAMP4

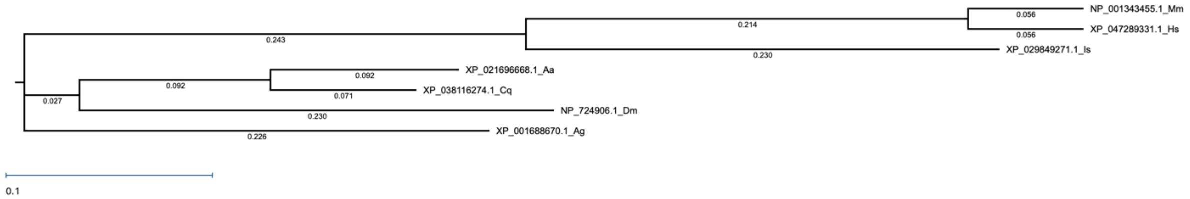

Supplementary Figure 4

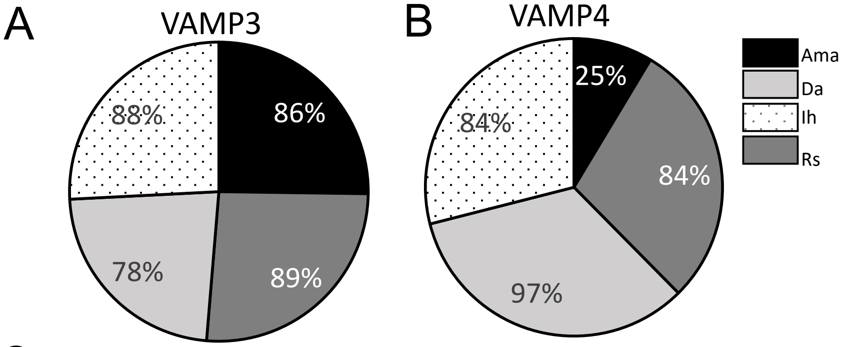

C

VAMP3			% Identity				
		Accession Number	A	B	C	D	E
% Similarity	A	KAK8769649.1_Ama		96.55	74.14	94.83	86.21
	B	XP_054921163.1_Da	96.55		76.72	98.28	88.79
	C	CAL6176945.1_Ih	75.86	78.45		75.86	77.59
	D	XP_049270500.1_Rs	94.83	98.28	77.59		87.93
	E	XP_029850929.1_Is	91.38	93.97	79.31	93.1	

D

VAMP4			% Identity				
		Accession Number	A	B	C	D	E
% Similarity	A	KAK8768612.1_Ama		20.14	25.9	20.86	25.18
	B	XP_050051708.1_Da	44.6		84.89	99.28	84.17
	C	CAL6182183.1_Ih	48.2	97.12		85.61	97.12
	D	XP_037524857.1_Rs	45.32	99.28	97.84		84.17
	E	XP_029849271.1_Is	48.2	96.4	99.28	97.12	

Supplementary Figure 5

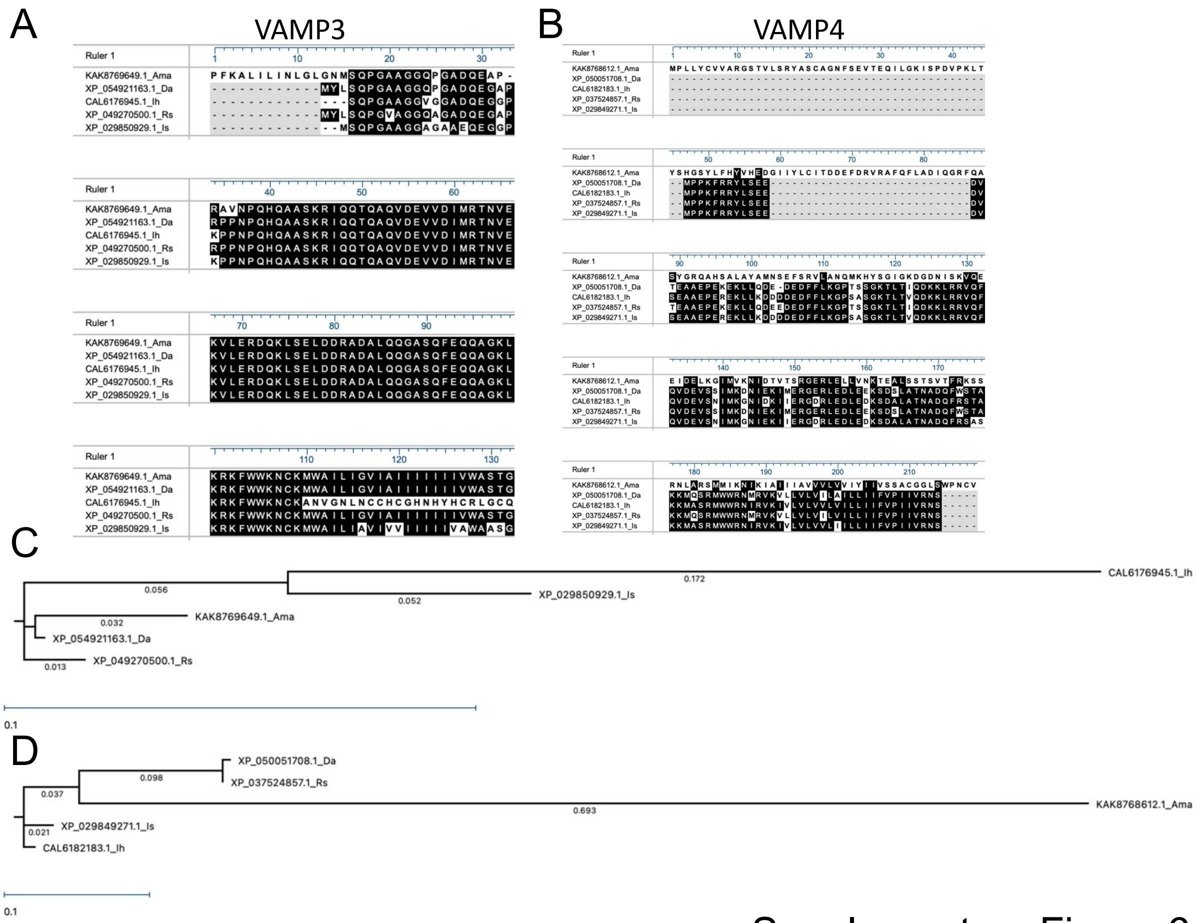

Supplementary Figure 6

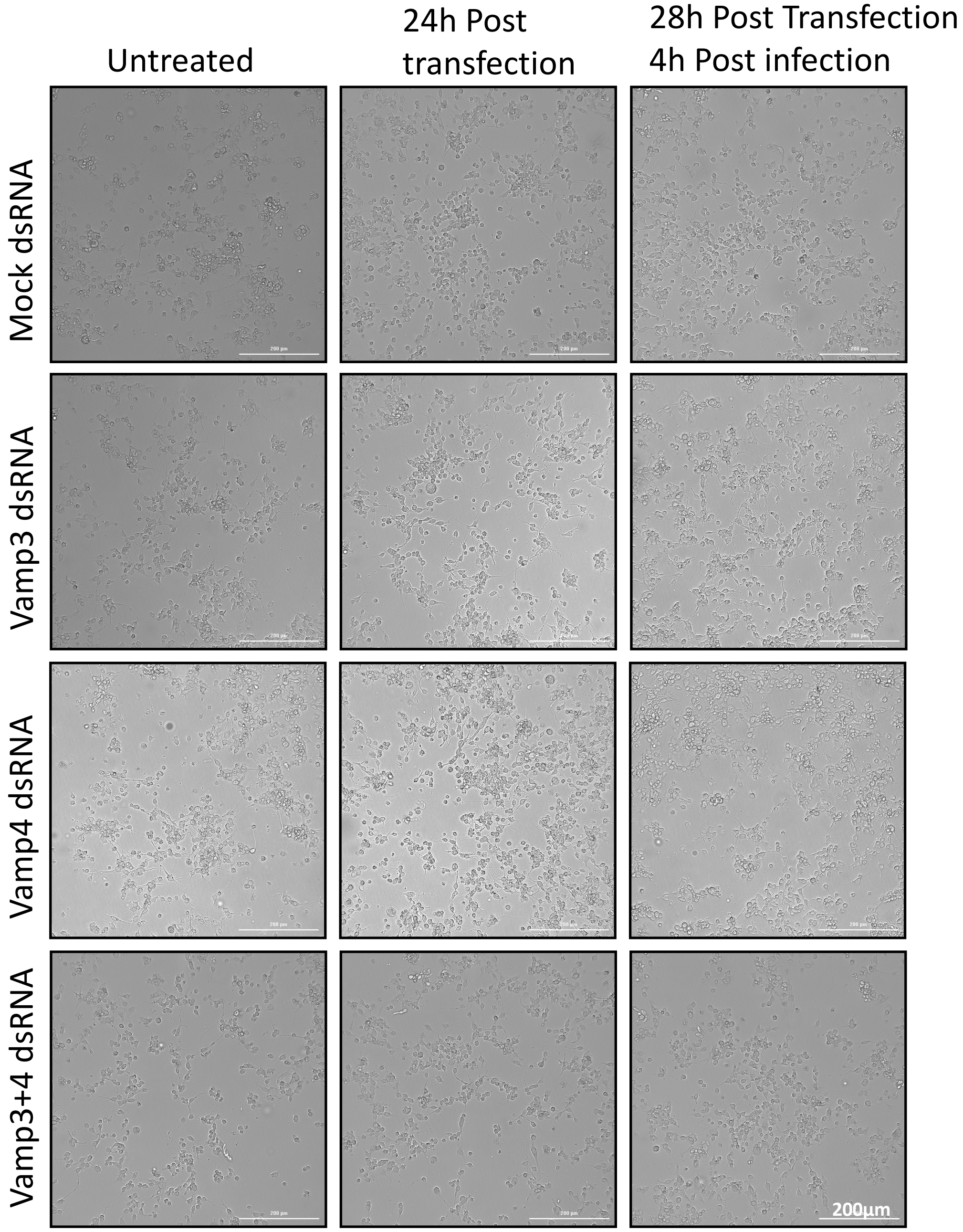

Supplementary Figure 7

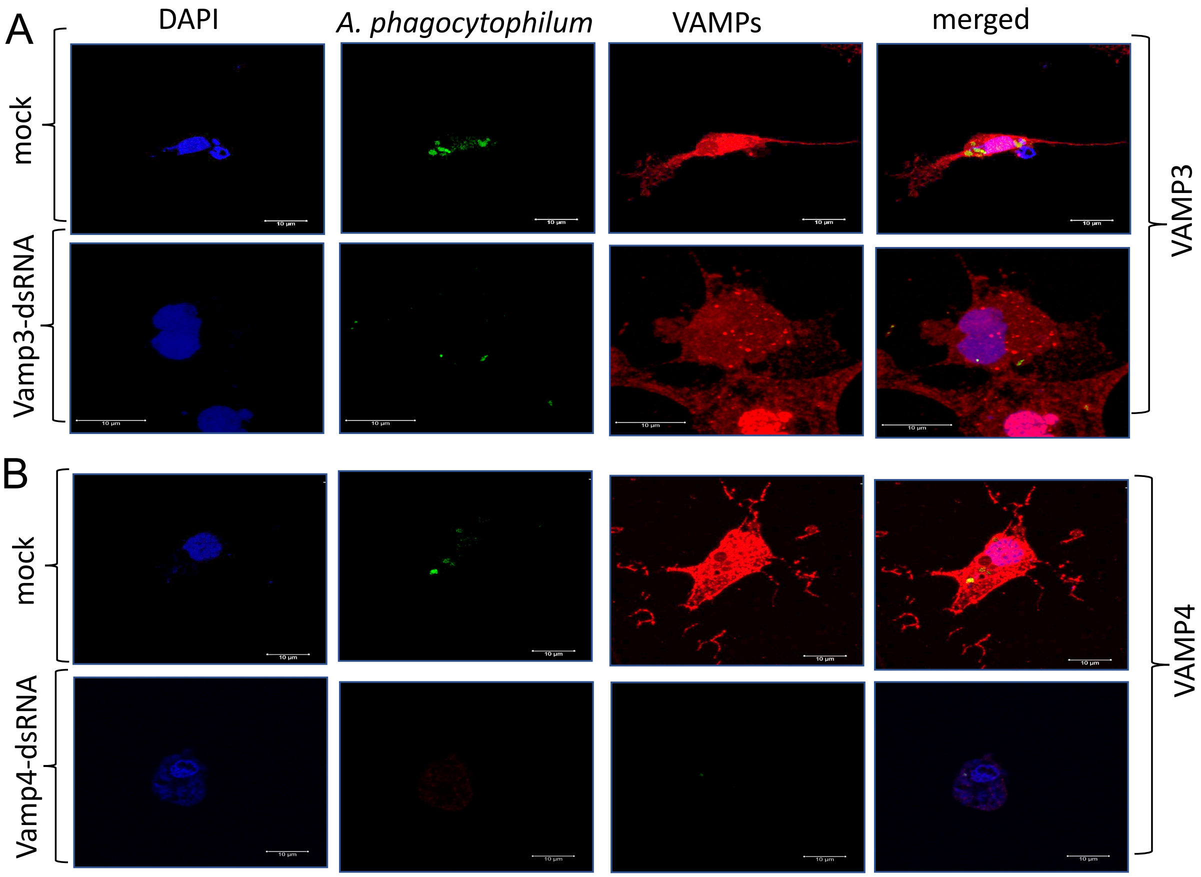

Supplementary Figure 8

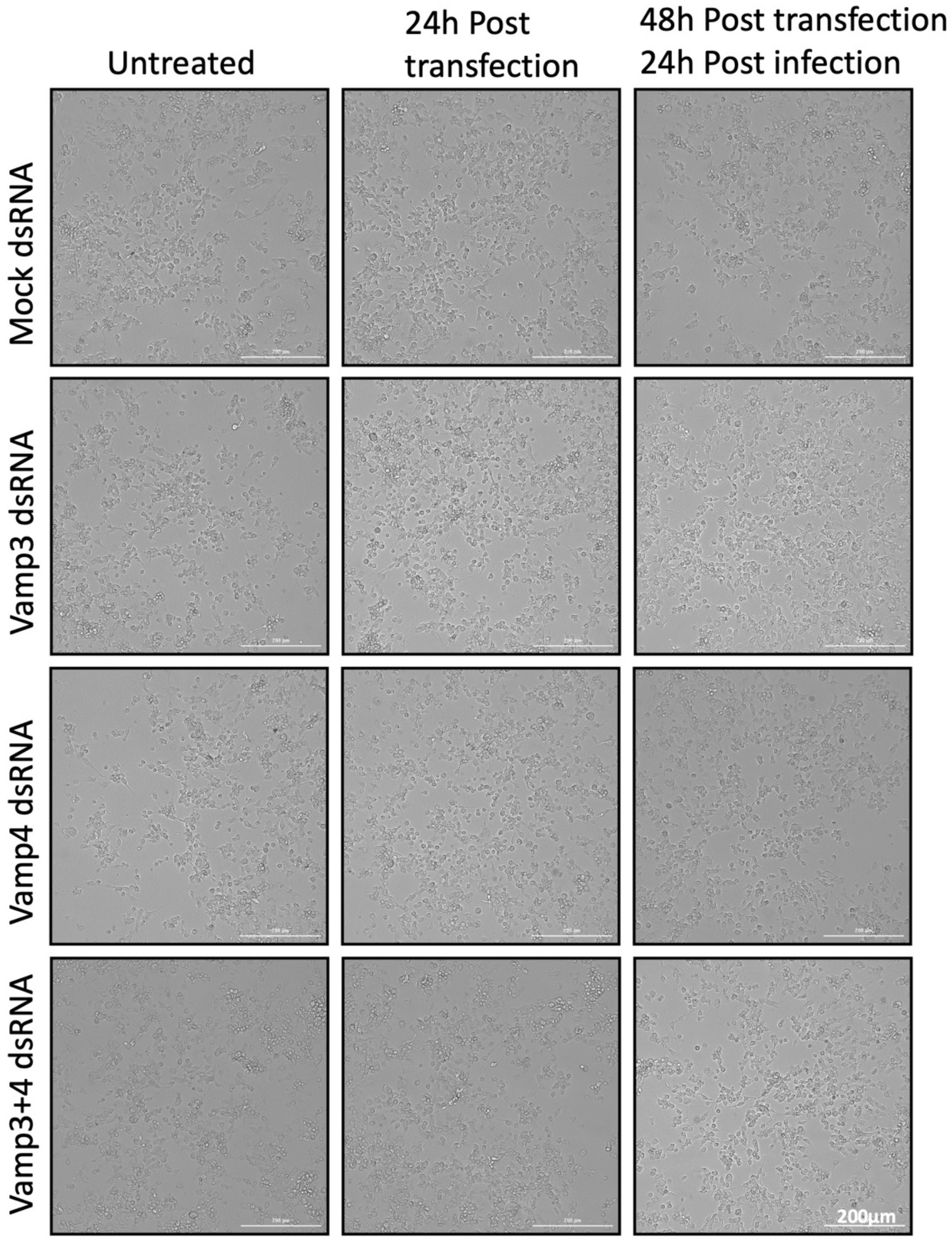

Supplementary Figure 9

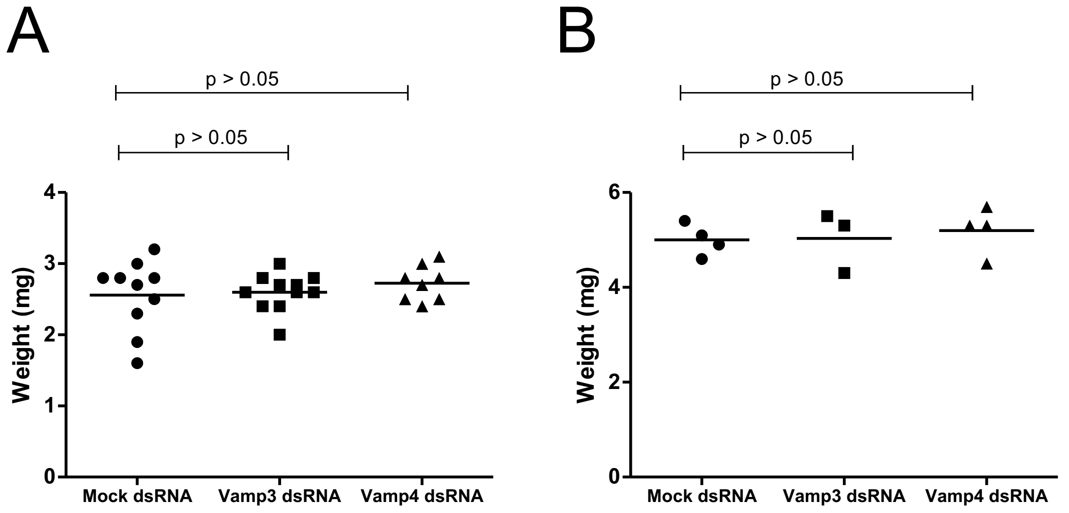

Supplementary Figure 10

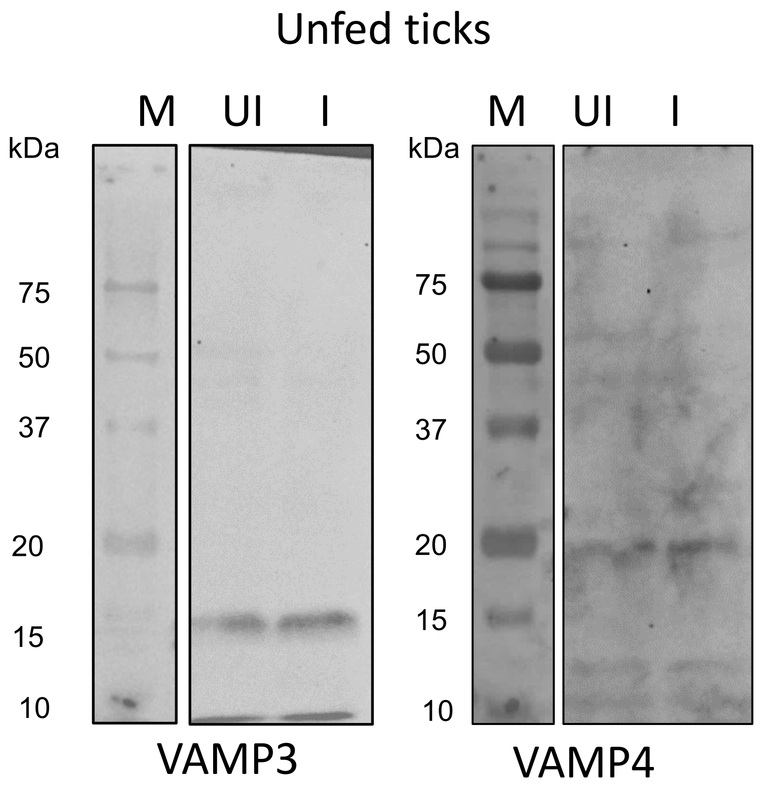

Supplementary Figure 11

Supplementary Table 1: Oligonucleotides used in this study.

Primer (5'-3')	Gene, purpose
GGTGGACGAGGTTGTGGATATCA	*vamp3* forward primer, QRT-PCR
GATAATGATTATGACCACAATGACAGCA	*vamp3* reverse primer, QRT-PCR
GGAAAGACACTCACGGTTCAAGA	*vamp4* forward primer, QRT-PCR
CCCTTATGTTACGCCACCACA	*vamp4* reverse primer, QRT-PCR
CCCCAGACGCCAGCTTGCA	*vamp7* forward primer, QRT-PCR
CCCTTCACGTCCTCCACCTGA	*vamp7* reverse Primer, QRT-PCR
TGAGATCTCTGTCGCCATCATTGATCCT	*vamp3* dsRNA forward primer, RNAi
CGGGTACCTTTCCACCAGAACTTCCTCTTC	*vamp3* dsRNA reverse primer, RNAi
TGAGATCTCATTAGCCACAAATGCTGACC	*vamp4* dsRNA forward primer, RNAi
CGGGTACCTGCAAGCCCTGCAGAAA	*vamp4* dsRNA reverse primer, RNAi
ACGAGGTTGTGGATATCATG	*vamp2* forward primer, QRT-PCR
GAACTTCCTCTTCAACTTGC	*vamp2* reverse primer, QRT-PCR
TCATACCTGAAGTTGTCCAA	*vamp33* forward primer, QRT-PCR
CTCAAGAATGCCACTGTTG	*vamp33* reverse primer, QRT-PCR
GCGGTTCATAAAGGAGTGGA	*dnaA* forward primer, QRT-PCR
AAACTTCCTGCGCCTCTGTA	*dnaA* reverse Primer, QRT-PCR
TCTAAGCGGTGGATCACTCGGT	*5.8S rRNA* forward primer, QRT-PCR
GACCCTCACACAGACGAAGCCA	*5.8S rRNA* reverse primer, QRT-PCR
CCAGCGTTTAGCAAGATAAGAG	*Anaplasma phagocytophilum p44 gene*
GCCCAGTAACAACATCATAAGC	*Anaplasma phagocytophilum p44 gene*

Supplementary Table 2. GenBank nucleotide accession numbers for *I. scapularis vamp* genes.

Gene	GenBank Accession Number	Organism
vamp3	XM_029995069.4	*Ixodes scapularis*
vamp4	XM_029993411.4	*Ixodes scapularis*
vamp7	XM_040505302.2	*Ixodes scapularis*
vamp2	EEC05846	*Ixodes scapularis*
vamp33	XM_002413880	*Ixodes scapularis*

Supplementary Table 3. GenBank accession numbers for proteins analyzed in this study.

Protein	Accession number	Organism
VAMP3	NP_004772.1	*Homo sapiens*
VAMP3	NP_033524.1	*Mus musculus*
VAMP3	NP_001261270.1	*Drosophila melanogaster*
VAMP3	XP_315508.4	*Anopheles gambiae*
VAMP3	XP_038116276.1	*Culex quinquifasciatus*
VAMP3	XP_02169669.1	*Aedes aegypti*
VAMP3	KAK8769649	*Amblyomma americanum*
VAMP3	XP_054921163	*Dermacentor andersoni*
VAMP3	CAL6176945	*Ixodes hexagonus*
VAMP3	XP_029850929.1	*Ixodes scapularis*
VAMP4	XP_047289331.1	*Homo sapiens*
VAMP4	NP_001343455.1	*Mus musculus*
VAMP4	NP_724906.1	*Drosophila melanogaster*
VAMP4	XP_001688670.1	*Anopheles gambiae*
VAMP4	XP_038116274.1	*Culex quinquifasciatus*
VAMP4	XP_021696668.1	*Aedes aegypti*
VAMP4	KAK8768612	*Amblyomma americanum*
VAMP4	XP_050051708	*Dermacentor andersoni*
VAMP4	CAL6182183	*Ixodes hexagonus*
VAMP4	XP_029849271.1	*Ixodes scapularis*
